# Tyrosinase Inhibitors With Enhanced Potency in Mammalian and Vertebrate Models, *N*‐(2‐Mercaptoethyl)benzamide Derivatives: Design, Synthesis, and Evaluation

**DOI:** 10.1111/cbdd.70383

**Published:** 2026-08-23

**Authors:** Hyunhee Ju, Minchang Kim, Hyunju Lee, Hee Jin Jung, Yeonsoo Jeong, Hyejin Kang, Hyeon Seo Park, Hae Young Chung, Hyung Ryong Moon

**Affiliations:** ^1^ Department of Manufacturing Pharmacy, College of Pharmacy and Research Institute for Drug Development Pusan National University Busan Republic of Korea; ^2^ Department of Pharmacy, College of Pharmacy Pusan National University Busan Republic of Korea

**Keywords:** kojic acid, melanin, *N*‐(2‐mercaptoethyl)benzamide, pigmentation, tyrosinase

## Abstract

Retaining structural features that allow 2‐mercaptomethylbenzimidazole compounds to function as Cu(II) chelators, *N*‐(2‐mercaptoethyl)benzamide (NMEB) derivatives **1a**–**1m** were designed, synthesized, and assessed for their potential as Cu(II) chelators. Derivative **1m** inhibited mushroom tyrosinase (mTYR) activity twice as potently as kojic acid (KA) in the presence of l‐DOPA. The mechanisms by which three derivatives, including **1m**, inhibit mTYR were elucidated through kinetic studies. Interestingly, all NMEB derivatives suppressed melanogenesis more potently than KA in B16F10 cells, significantly reducing melanin production in a concentration‐dependent manner. NMEB derivatives also inhibited cellular TYR activity in keeping with their melanogenesis inhibition, indicating B16F10 cellular TYR inhibition to be the primary mechanism. Moreover, among these derivatives, **1a** inhibited zebrafish larval pigmentation hundreds of times more potently than KA. In mammalian cells and zebrafish larvae, NMEB derivatives showed even higher TYR‐inhibitory or antimelanogenesis potency than mushrooms. Due to differences in the amino acid sequence and location of TYRs, NMEB derivatives appear to differ in their inhibition of TYR activity and melanin production in different species. This study points out limitations in the development of novel TYR inhibitors based on mTYR inhibition results and provides fundamental data supporting their development as novel skin‐lightening agents.

## Graphical abstract

*N*‐(2‐mercaptoethyl)benzamide derivatives exhibited a much stronger tyrosinase inhibitory effect in higher organisms than in lower organisms. These derivatives exerted a much more potent anti‐melanogenic effect in zebrafish larvae and mammalian cells than in mushrooms.

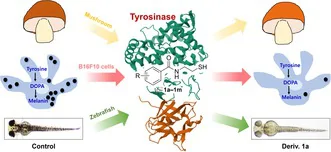

## 1 Introduction

Tyrosinase (TYR) is a polyphenol oxidase that oxidizes *ortho*‐diphenols and monophenols to *ortho*‐quinones and *ortho*‐diphenols, respectively, and is involved in the series of melanin synthesis processes constituting melanogenesis (Ito et al. 2020; Ramsden and Riley 2014). TYR catalyzes the rate‐determining first two steps of melanogenesis. TYR is thus a key target for melanogenesis regulation. Diverse compounds capable of modulating TYR activity have been identified as potential medicines for treating pigment‐related disorders and cosmetics for skin‐lightening (Kang et al. 2025). Hydroquinone, kojic acid (KA), arbutin, aloesin, resorcinol, l‐ascorbic acid, flavonoids, tranexamic acid, and analogs of these compounds are known to inhibit TYR (Lv et al. 2025; Peng et al. 2023; Rezapour Niri et al. 2023; Roulier et al. 2023; Xie et al. 2024). Recently, synthetic TYR inhibitors that display potential for greater efficacy and safety than natural inhibitors have been actively developed (Ashooriha et al. 2020; Ullah et al. 2019).

TYR inhibitors are used in the cosmetics industry primarily to whiten skin and prevent pigmentation, and demand for these products is growing rapidly, particularly in the Asia‐Pacific region (Zolghadri et al. 2019). Although existing inhibitors, including hydroquinone, KA, and arbutin, are effective, adverse effects and safety concerns have increased the need for alternatives (Pillaiyar et al. 2017). The objective of this study was to discover new TYR inhibitors and provide fundamental data that can contribute to the development of skin‐lightening products and improve TYR inhibitor efficacy. We designed and synthesized new TYR inhibitor candidates, evaluated their inhibitory activity against various TYRs, assessed their ability to inhibit melanin production in biological systems, and elucidated their inhibitory mechanisms.

**FIGURE 1:**
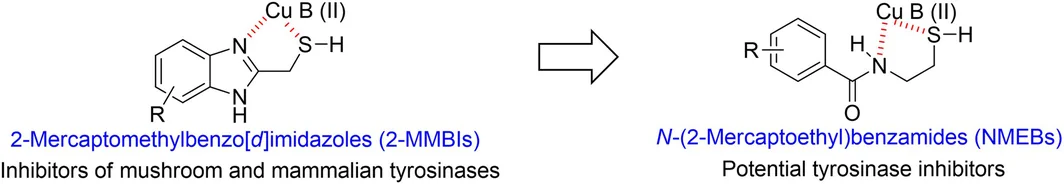
Illustration of Cu(II) chelation by 2‐mercaptomethylbenzo[*d*]imidazoles (2‐MMBIs) known to inhibit mushroom and mammalian TYRs and a plausible model of Cu(II) chelation by *N*‐(2‐mercaptoethyl)benzamides (NMEBs).

In Cu(II)‐chelating ability experiments performed using pyrocatechol violet, a Cu(II)‐chelating agent, and CuSO_4_, several 2‐mercaptomethylbenzimidazoles (2‐MMBIs; Figure 1), in which the nitrogen and sulfur atoms are separated by two carbon atoms, exhibited copper‐chelating abilities (Buitrago et al. 2014; Jung et al. 2024). 2‐MMBIs also inhibited mushroom TYR (mTYR), B16F10 cellular TYR (cTYR), and zebrafish larval pigmentation, owing to their Cu(II)‐chelating capacity. Based on these previous findings, *N*‐(2‐mercaptoethyl)benzamide (NMEB) derivatives containing nitrogen and sulfur atoms that are separated by two carbon atoms, such as 2‐MMBIs, were designed as candidate TYR inhibitors (Figure 1). Docking simulation results, obtained prior to the synthesis of NMEB derivatives, supported their potential as TYR inhibitors, driven by hydrogen bonding via NH and SH groups alongside hydrophobic interactions of the aryl moiety. NMEB derivatives were thus synthesized, and their biological activities associated with regulation of melanin production were evaluated. The inhibitory mechanisms of the functional derivatives were identified.

## 2 Results and Discussion

### 2.1 Design of NMEB Derivatives

Thirteen NMEB derivatives were designed for assessment of TYR inhibitory capability: *N*‐(2‐mercaptoethyl)benzamide (**1a**) and twelve *N*‐(2‐mercaptoethyl)benzamides substituted on the phenyl ring (PR) (2‐, 3‐, and 4‐methyl‐substituted NMEB derivatives [**1b**, **1c**, and **1d**]; 3‐fluoro‐, 3‐chloro‐, and 3‐bromo‐2‐NMEB derivatives [**1e**, **1f**, and **1g**]; 4‐fluoro‐, 4‐chloro‐, and 4‐bromo‐2‐MMBI derivatives [**1h**, **1i**, and **1j**]; 3,4‐dichloro‐ and 3,4‐dimethoxy‐2‐NMEB derivatives [**1k** and **1l**]; and 3,4,5‐trimethoxy‐2‐NMEB derivative [**1m**]). Excluding **1a**, among the remaining 2‐NMEB derivatives, **1e**–**1k** carried electron‐withdrawing groups (EWGs) on the PR, and derivatives **1b**–**1d**, **1l**, and **1m** carried electron‐releasing groups (ERGs).

### 2.2 Docking Simulation of NMEB Derivatives at the mTYR Active Site (ActS)

The probability that the designed NMEB derivatives would bind well to the TYR ActS was investigated using *in silico* docking simulations. The X‐ray crystal structure coordinates of mTYR (*Agaricus bisporus*) stored in the Protein Data Bank were used for docking simulations performed in Chimera 1.19 (https://www.cgl.ucsf.edu/chimera/download.html) with AutoDock Vina 1.2.0 (The Scripps Research Institute, La Jolla, San Diego, CA, USA) software (Figure **S79**). Derivatives **1h** and **1k**, with an EWG, and derivative **1m**, with an ERG, were subjected to docking simulations after preparation of energy‐minimized three‐dimensional structures using Chem3D Pro 12.0. We investigated chemical interactions of NMEB derivatives in the mTYR ActS with LigandScout 4.4.8.

**FIGURE 2:**
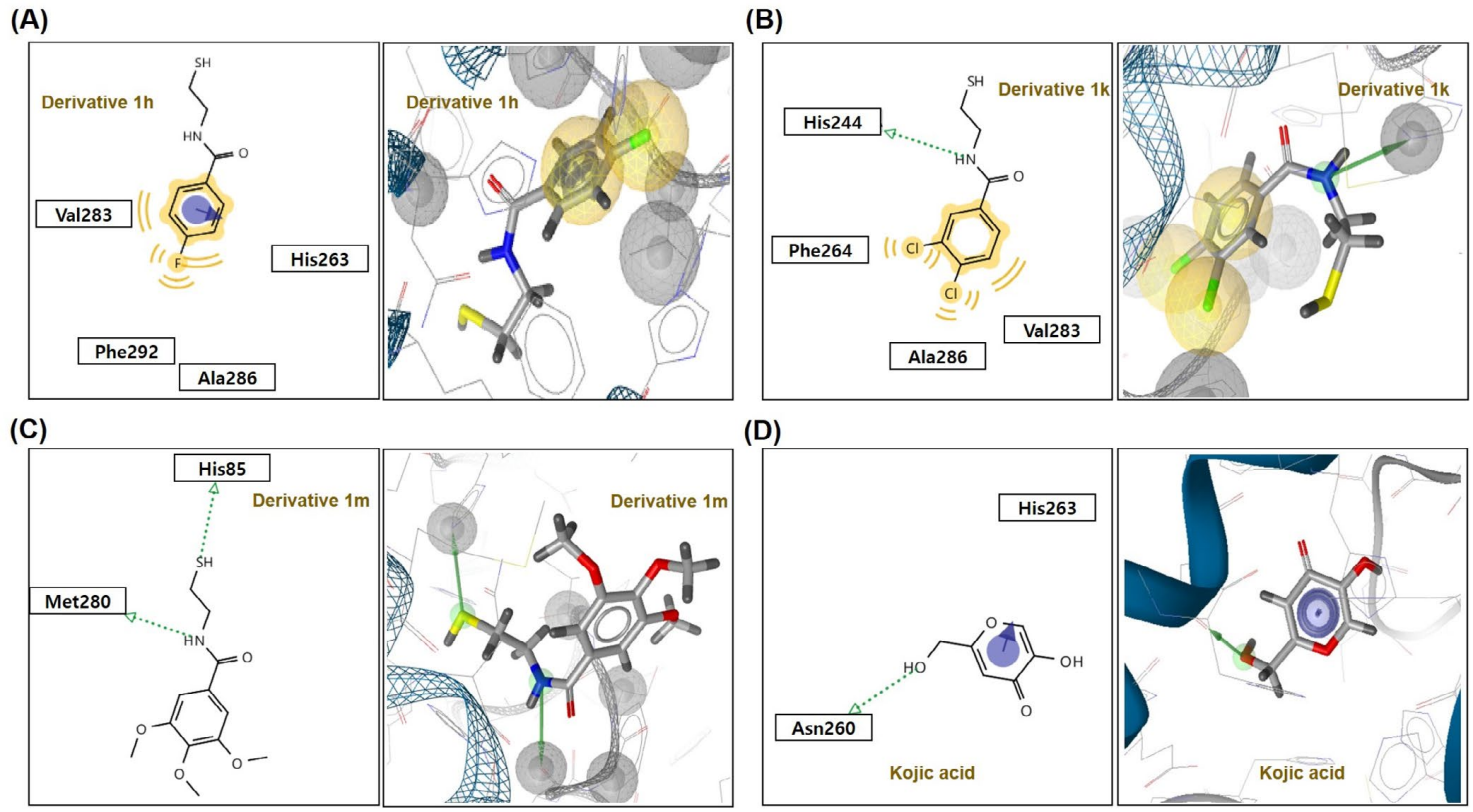
Illustrated docking simulations of mTYR ActS with NMEB derivatives **1h** (A), **1k** (B), and **1m** (C) and KA (D). Green arrows, yellow waves, and blue arrows represent H‐bonds, HP interactions, and pi‐pi stacking interactions, respectively.

The ActS binding affinities of NMEB derivatives **1h**, **1k**, and **1m** were compared with that of KA (positive control) (Lin et al. 2025). Four amino acid residues in mTYR ActS interacted with derivative **1h** (Figure 2). The PR made hydrophobic (HP) interactions with Ala286 and Val283. The 4‐fluorine also created HP interactions with Val283 and Phe292. Additionally, **1h** formed a pi‐pi stacking interaction with His263. Derivative **1k** also interacted with four amino acids. Both chlorines formed HP interactions with Val283, with the 3‐ and 4‐chlorines interacting with Phe264 and Ala286, respectively. The hydrogen on the amidic nitrogen hydrogen‐bonded to His244 as a hydrogen bond donor (HBD). Unlike **1h** and **1k**, each bearing an EWG, **1m**, bearing an ERG, only participated in hydrogen bonding (H‐bonding) with no HP interactions. Two H‐bonds were formed to His85 and Met280, with the hydrogen atoms of the mercapto and amido groups serving as HBDs. Val283, Ala286, His263, Phe264, His244, Phe292, His85, and Met280, which interact with NMEB derivatives, are frequently targeted by TYR inhibitors (Azimi et al. 2024; Jewgiński et al. 2025; Mohd Fahmi et al. 2025; Wavhal et al. 2025). The alcoholic hydroxyl group of KA interacts with Asn260 via an H‐bond, and the pyran‐4‐one ring interacts with His263 via pi‐pi stacking. These interactions provide derivatives **1h**, **1k**, and **1m** with respective binding energies of −5.9, −5.7, and −5.0 kcal/mol and KA with a binding energy of −5.4 kcal/mol. Although docking simulations did not reveal Cu(II)‐chelation by **1h**, **1k**, and **1m**, because derivatives **1h** and **1k** showed stronger binding affinities than KA, we sought to synthesize NMEB derivatives and examine their TYR inhibitory activity.

### 2.3 Synthesis of NMEB Derivatives

**SCHEME 1:**
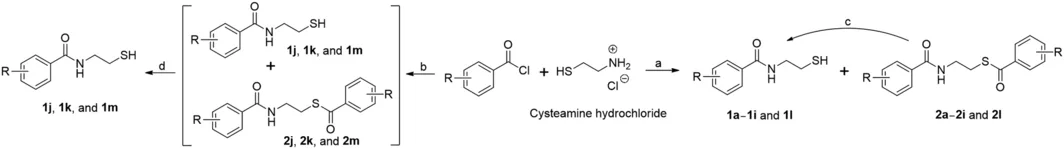
Synthesis of *N*‐(2‐mercaptoethyl)benzamide derivatives (**1a**–**1m**). Reagents and conditions: (a) Et_3_N, DCM, 15°C–17°C, 5–10 h, 35%–54% for **1a**–**1i** and **1l**, and 24%–42% for **2a**–**2i** and **2l**; (b) Et_3_N, DCM, 15°C–17°C, 5–7 h; (c) K_2_CO_3_, MeOH, 15°C–17°C, 5–10 h, 79%–97%; (d) K_2_CO_3_, MeOH, 15°C–17°C, 7–12 h, 68%–81% (2‐step yield).

NMEB derivatives **1a**–**1m** were synthesized by reacting benzoyl chloride derivatives with cysteamine hydrochloride. Condensation of various benzoyl chlorides with cysteamine hydrochloride in the presence of triethylamine yielded NMEB derivatives **1a**–**1m** and *S*‐(2‐(benzamido)ethyl)benzothioate derivatives **2a**–**2m** (Scheme 1). NMEB derivatives **1a**–**1i** and **1l** and benzothioate derivatives **2a**–**2i** and **2l** were initially separated using column chromatography. The benzothioate derivatives (**2a**–**2i** and **2l**) were hydrolyzed with potassium carbonate in methanol to yield additional NMEB derivatives (**1a**–**1i** and **1l**). Thereafter, NMEB derivatives (**1j**, **1k**, and **1m**) were obtained by condensation of benzoyl chlorides with cysteamine followed by direct hydrolysis of **2j**, **2l**, and **2m** with potassium carbonate in methanol without purification. NMEB derivative structures were elucidated using ^1^H and ^13^C nuclear magnetic resonance (NMR) spectra (Figures S1–S49). NMEB derivatives **1a**–**1m** were commercially available or known compounds (Armendarez et al. 2024; El‐Gendy et al. 2013; Mano et al. 2009; Marichev et al. 2017; Petersson et al. 2009).

### 2.4 mTYR Inhibitory Activity

The capacity of the NMEB derivatives to inhibit TYR activity was assessed using mTYR as the test enzyme. l‐DOPA and l‐tyrosine were used as respective substrates of mTYR's diphenolase and monophenolase activities in inhibition experiments. For both substrates, three test sample concentrations (4, 20, and 100 μM) were employed to calculate their IC_50_ values. Each compound's mTYR inhibitory activities were compared with those of KA (positive control).

KA and NMEB derivatives **1a**–**1m** IC_50_ values are displayed in Table 1 (Figures S50–S59). When using l‐tyrosine as a substrate, NMEB derivatives with no substitution on the PR (**1a**), a methyl group on the PR (**1b**–**1d**), or a fluorine at position 3 of the PR (**1e**) all yielded IC_50_ values exceeding 300 μM. In contrast, the presence of chlorine at positions 3 or 4 on the PR (**1f** and **1i**) or at positions 3 and 4 on the PR (**1k**) yielded IC_50_ values ranging from 45 to 59 μM (3–4 times higher than that of KA (IC_50_ value: 14 ± 2 μM)). Derivative **1h**, with a 4‐fluorophenyl, and derivative **1m**, with a 3,4,5‐trimethoxyphenyl, revealed moderate inhibition, with respective IC_50_ values of 47 ± 4 and 86 ± 3 μM. Derivatives **1g**, **1j**, and **1l**, respectively containing a 3‐bromophenyl, a 4‐bromophenyl, or a 3,4‐dimethoxyphenyl group, showed weak inhibitory potency, with IC_50_ values ranging from 200 to 250 μM. NMEB derivatives **1f**, **1h**, **1i**, **1k**, and **1m** exhibited moderate mTYR inhibitory activity, but none outperformed KA in inhibiting mTYR activity.

**TABLE 1:** IC_50_ values of NMEB derivatives **1a**–**1m** against mTYR in the presence of l‐tyrosine or l‐DOPA.

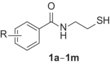			
Compound	*R*	IC_50_ (μM, mean ± SEM^a^)	
		l‐Tyrosine	l‐DOPA
**1a**	H	> 300	> 300
**1b**	*o*‐Methyl	> 300	> 300
**1c**	*m*‐Methyl	> 300	> 300
**1d**	*p*‐Methyl	> 300	> 300
**1e**	*m*‐Fluoro	> 300	> 300
**1f**	*m*‐Chloro	59 ± 3	70 ± 2
**1 g**	*m*‐Bromo	240 ± 30	60 ± 4
**1 h**	*p*‐Fluoro	47 ± 4	40.0 ± 0.8
**1i**	*p*‐Chloro	45 ± 4	230 ± 14
**1j**	*p*‐Bromo	250 ± 30	107 ± 7
**1 k**	3,4‐Dichloro	49 ± 1	35 ± 3
**1 l**	3,4‐Dimethoxy	200 ± 20	76 ± 1
**1 m**	3,4,5‐Trimethoxy	86 ± 3	10 ± 1
Kojic acid^b^		14 ± 2	21 ± 1

^a^ Standard error of the mean.

^b^ Positive control. IC_50_ values above 100 μM were obtained by extrapolation.

When using l‐DOPA as a substrate, NMEB derivatives with no substitution on the PR (**1a**), a methyl group on the PR (**1b**–**1d**), or a fluorine at position 3 of the PR (**1e**) all yielded IC_50_ values exceeding 300 μM, similar to their mTYR inhibition with l‐tyrosine as a substrate. Derivatives **1i** and **1j**, containing a 4‐chlorophenyl and a 4‐bromophenyl, respectively, revealed relatively weak mTYR inhibition, with respective IC_50_ values of 230 ± 14 and 107 ± 7 μM. NMEB derivatives **1f**–**1h**, **1k**, and **1l**, containing a 3‐chloro, a 3‐bromo, a 4‐fluoro, a 3,4‐dichloro, and a 3,4‐dimethoxy group on the PR, respectively, moderately inhibited mTYR, with IC_50_ values ranging from 35 to 76 μM (1.6–3.6‐fold higher than that of KA (IC_50_ value: 21 ± 1 μM)). The most potent inhibitory activity was obtained with derivative **1m** with a 3,4,5‐trimethoxy on the PR. Its IC_50_ value was 10 ± 1 μM, 2.1‐fold lower than that of KA.

**FIGURE 3:**
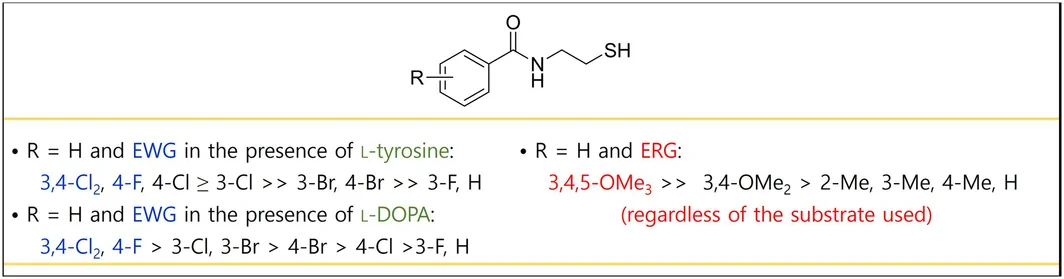
Relationships between PR substituent types on NMEB derivatives and mTYR inhibitory activity in the presence of l‐DOPA and l‐tyrosine.

The structure–activity relationships (SAR) between the NMEB derivatives and mTYR are summarized in Figure 3. When EWG substituents were placed on the PR, derivatives with EWGs (chlorine and fluorine) at PR position 4 or positions 3 and 4 most strongly inhibited mTYR, whereas derivatives that had fluorine at PR position 3 or that lacked a substituent exhibited the lowest mTYR inhibitory activity. These results were independent of the substrate used. In compounds with ERG substituents on their PR, stronger ERGs produced stronger inhibitory effects (OMe > > Me, H), regardless of substrate used. Greater ERG numbers present on the PR also increased inhibitory activity (3,4,5‐trimethoxy >>> 3,4‐dimethoxy).

### 2.5 Inhibitory Mechanism Study

NMEB derivatives **1h**, **1k**, and **1m**, which showed 0.53–2.1 times potency in mTYR inhibition compared to KA in the presence of l‐DOPA, were used to study their inhibitory mechanism. To obtain kinetic data for mTYR in the presence of NMEB derivatives, the substrate (l‐DOPA) was assayed at 1, 2, 4, 8, and 16 mM, with NMEB derivatives included at 0, 12.5, 25, or 50 μM for **1h** and **1k** and at 0, 5, 10, or 20 μM for **1m**.

**FIGURE 4:**
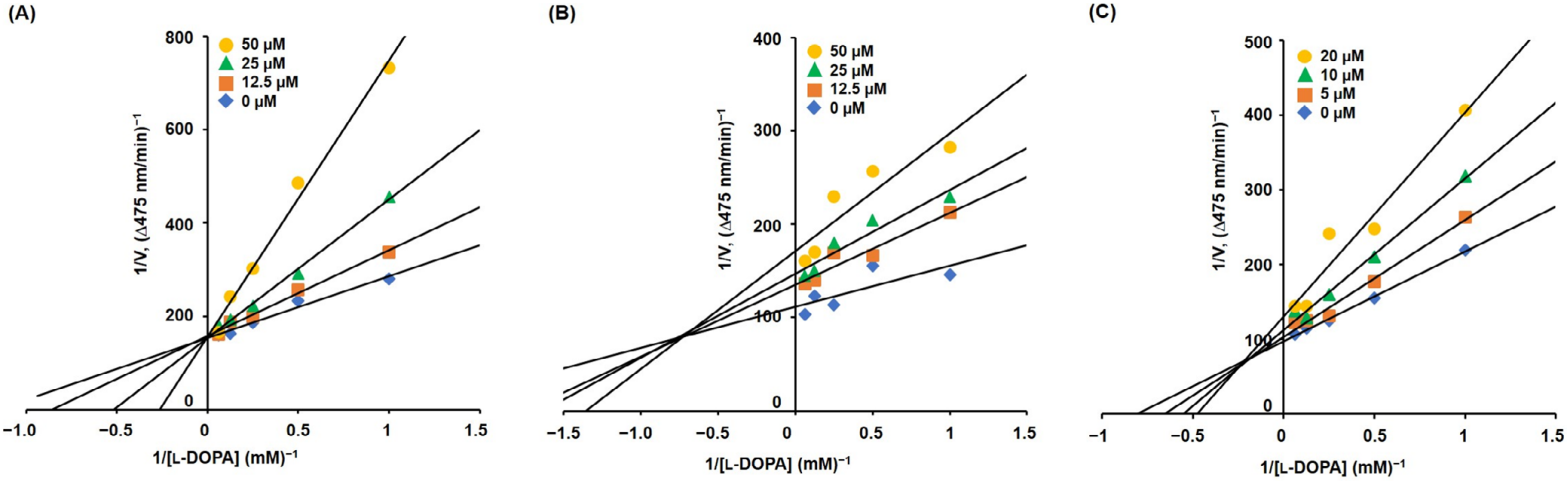
Lineweaver‐Burk plots of derivatives **1h** (A), **1k** (B), and **1m** (C) obtained from kinetic experiments. Four concentrations (0, 12.5, 25, and 50 μM for **1h** and **1k**; 0, 5, 10, and 20 μM for **1m**) of NMEB derivatives were used, each with five l‐DOPA concentrations (16, 8, 4, 2, and 1 mM).

Lineweaver‐Burk plots for each derivative were prepared from the kinetic data and are shown in Figure 4. Each plot displays four straight lines with progressively steeper slopes as NMEB derivative concentration increases. These lines converge at one point on the y‐axis for **1h** (Jan et al. 2025) or in the second quadrant for **1k** and **1m** (Du et al. 2026), indicating that **1h** is a competitive mTYR inhibitor and **1k** and **1m** are mixed‐type mTYR inhibitors. These results imply that derivatives **1k** and **1m** can bind to both mTYR's active and allosteric sites.

To determine NMEB derivative affinities for mTYR, Dixon plots for each derivative were prepared from the kinetic data used to acquire the Lineweaver‐Burk plots. Dixon plots for each derivative are displayed in Figure S1. These plots display four straight lines with increasing slopes as substrate concentration decreases. The straight lines in each plot converge at a point in the second quadrant. Inhibition constants (K_i_) for each derivative were obtained from the convergence points in the Dixon plots. The K_i_ values for the NMEB derivatives **1h**, **1k**, and **1m** were 29.9 × 10^−3^, 29.1 × 10^−3^, and 13.0 × 10^−3^ mM, respectively. Smaller K_i_ values indicate stronger binding between enzyme and inhibitor (Nazir et al. 2021). Analog **1m**, which inhibited mTYR activity toward l‐DOPA more potently than **1h** and **1k**, had a smaller K_i_ value than did analogs **1h** and **1k**. Similar results were observed with methoxy‐substituted tyramine derivatives (Nazir et al. 2021) and (*Z*)‐4‐(substituted benzylidene)‐2‐phenyl‐1*H*‐imidazol‐5(4*H*)‐ones (Jung, Park, Jeong, Kang, et al. 2026).

### 2.6 Docking Simulations of NMEB Derivatives in the mTYR Allosteric Site

The results of inhibition mechanism studies obtained through kinetic experiments indicate that NMEB derivatives **1k** and **1m** are mixed‐type inhibitors that can also bind to mTYR's allosteric site. Docking simulations of NMEB derivatives **1k** and **1m** with mTYR were thus performed to validate binding of these derivatives to allosteric sites. Chem3D Pro 12.0, ChemDraw Ultra 12.0, Chimera 1.19, AutoDock Vina 1.2.0, and LigandScout 4.4.8 were used for these allosteric site docking simulations, as in the ActS docking simulations.

The binding affinities of NMEB derivatives **1k** and **1m** at the allosteric site were −5.9 and −5.5 kcal/mol, respectively. These two derivatives are bound into the same allosteric position, as shown in Figure S2A,B. Derivatives **1k** and **1m** are represented in yellow and red, respectively. Derivative **1k** formed two H‐bonds and two HP interactions. The oxygen and amino (NH) groups of the amide functional group acted as respective H‐bond acceptors (HBA) and HBD in interactions with Gln307 and Glu356, and the 3,4‐dichlorophenyl ring interacted with Thr308 and Trp358 via HP interactions. In contrast, derivative **1m** served only as an HBA via the oxygen of its 3‐methoxy group. The allosteric binding sites of derivatives **1k** and **1m** precisely match the binding sites of *p*‐anisaldehyde, a volatile component of loquat honey with TYR inhibitory activity (Zhang et al. 2025). In docking simulations, *p*‐anisaldehyde interacted with Trp358, Glu356, Thr308, and Gln307, as do derivatives **1k** and **1m**. The binding energies of these derivatives indicate that **1k** and **1m** bind more strongly to the allosteric site than KA binds to the ActS. Considering that derivative **1 m** binds to the ActS with a binding energy of −5.0 kcal/mol, these results suggest that **1m** binds more strongly to the allosteric site than to the ActS.

### 2.7 Cu(II)‐Chelating Capacity

According to the docking simulation results, derivative **1m** had a weaker binding affinity for mTYR ActS than KA and bound more strongly to the allosteric site than the ActS. No chelation of Cu(II) ions was observed in docking simulations of NMEB derivatives with the mTYR ActS. These findings raised the question of whether NMEB derivatives could chelate Cu(II) ions present in the ActS of mTYR. CuSO_4_ and pyrocatechol violet were thus used as a Cu(II) source and a chelating agent, respectively, to examine the Cu(II)‐chelating capacity of the NMEB derivatives (Saiga et al. 2003; Santos et al. 2017). Pyrocatechol violet‐Cu(II) complexes form a bluish‐violet color. After pyrocatechol violet was mixed with CuSO4 in the presence of NMEB derivatives, absorbance at 632 nm was measured to determine Cu(II)‐chelating capacity (Su et al. 2010).

KA, used as a positive control, showed 32% Cu(II)‐chelating capacity (Figure S60). Among the NMEB derivatives, **1k** was the best Cu(II) chelator, but it only chelated 7%. The other derivatives chelated less than 5%. Contrary to our expectations, all NMEB derivatives exhibited very weak Cu(II)‐chelating capacity, and Cu(II)‐chelating interactions of NMEB derivatives with mTYR were not observed in docking simulations. NMEB derivatives lacked Cu(II)‐chelating capacity despite having a structure similar to that of 2‐MMBIs (Figure 1) (Jung et al. 2024). This is likely due to the lack of electron density on the nitrogen atom caused by the electron‐withdrawing effect of the adjacent carbonyl group in the NMEB derivative structure (Figure 1). Despite the lack of Cu(II)‐chelating capacity, **1m** displayed mTYR inhibitory activity 2.1‐fold stronger than KA. Additionally, several NMEB derivatives exhibited moderate mTYR inhibitory activity. Several studies have demonstrated that compounds with weak to moderate mTYR inhibitory activity can also possess excellent TYR inhibitory activity and melanin production inhibition activity in B16F10 cell experiments (Gaweł‐Bęben et al. 2023; Jung, Park, Jeong, Kim, et al. 2026). We thus investigated whether NMEB derivatives could also block mammalian B16F10 cTYR activity.

### 2.8 Melanin Production Inhibition in B16F10 Cells

Prior to assessing B16F10 cTYR inhibition, we evaluated the potency of the NMEB derivatives in inhibiting melanin synthesis in B16F10 cells to select NMEB derivatives for an in‐depth B16F10 cTYR inhibition experiment. B16F10 cells were exposed to stimulators (Note: The stimulators used in all experiments in this paper comprised IBMX [3‐isobutyl‐1‐methylxanthine; 200 μM] and α‐MSH [α‐melanocyte‐stimulating hormone; 1 μM]) 1 h after exposure to 20 μM test compounds (NMEB derivatives **1a**–**1m** and KA [positive control]) and incubated for 72 h. By measuring absorbance at 405 nm, we quantitated the melanin inhibitory effect of **1a**–**1m**.

**FIGURE 5:**
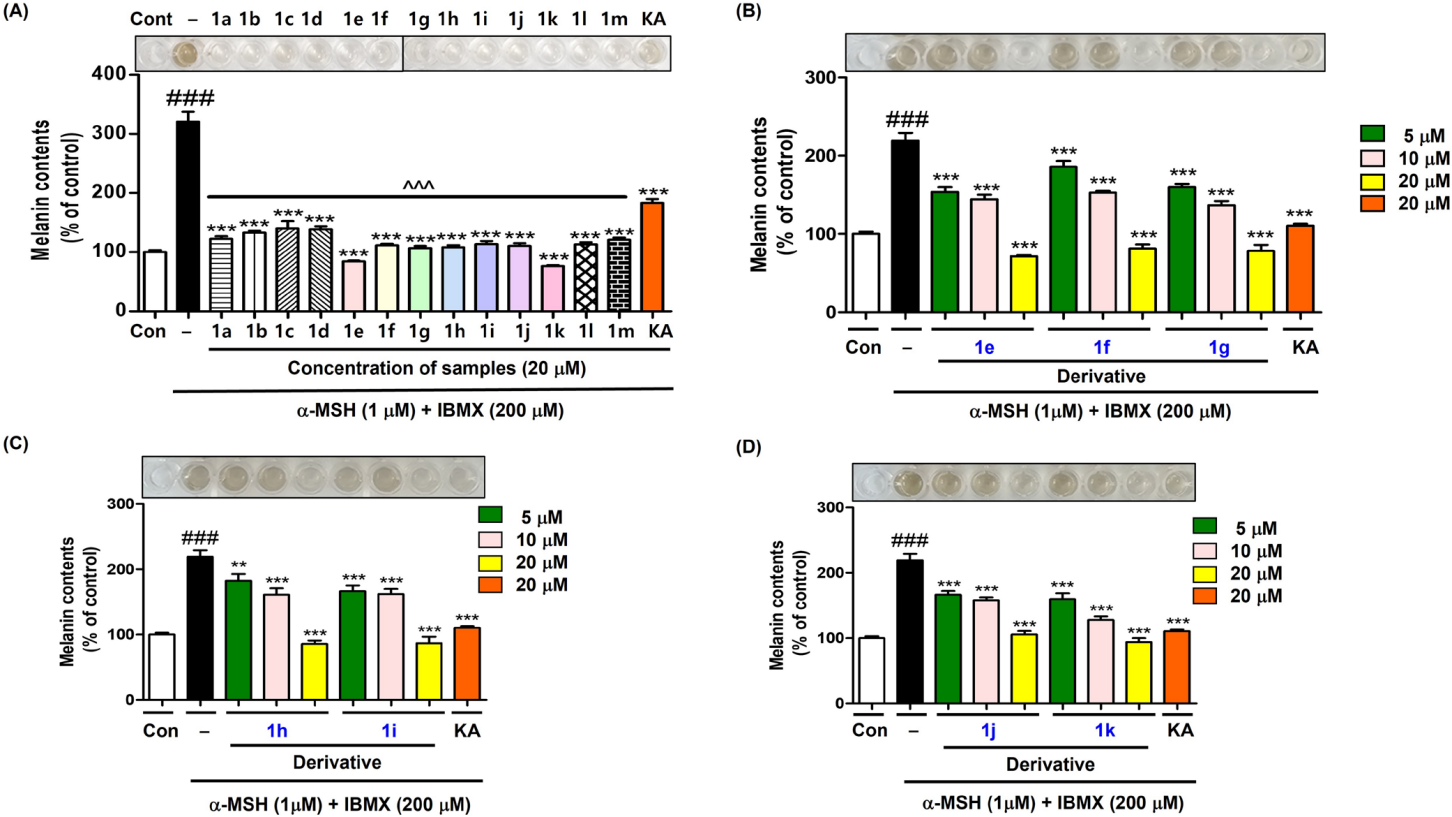
Melanin content in the presence of NMEB derivatives **1a**–**1m** (20 μM) (A) and **1e**–**1k** (5, 10, and 20 μM) (B–D) in B16F10 cells. Cells were exposed to test samples for 1 h before stimulator treatment with α‐MSH (α‐melanocyte‐stimulating hormone) and IBMX (3‐isobutyl‐1‐methylxanthine) at 1 and 200 μM, respectively, for 72 h. Melanin content was expressed as a percentage relative to untreated cells. The melanin inhibitory effect was compared to that obtained with KA (20 μM; positive control). **p < 0.01 and ***p < 0.001 vs. group treated with stimulator; ^###^ *p* < 0.001 vs. control; ^^^^^ *p* < 0.001 vs. group treated with KA.

Stimulator treatment raised melanin content, and KA treatment significantly ameliorated this stimulator‐induced increase. Unlike mTYR inhibitory activity, all derivatives inhibited melanin production significantly more potently than KA (Figure 5A and Figure S61). The same results were observed for *N*‐benzylthiocarbamate analogs (Jung, Park, Jeong, Kim, et al. 2026). The *N*‐benzylthiocarbamate analogs potently inhibited melanogenesis despite low mTYR inhibitory activity. To assess the concentration dependence of inhibition of melanin production, derivatives **1e**–**1k**, which most potently inhibited melanin production, were chosen. All derivatives chosen were tested at 5, 10, and 20 μM and showed concentration‐dependent inhibitory activity (Figure 5B–D and Figure S62). At 20 μM, all derivatives inhibited melanin production more potently than 20 μM KA. To investigate whether inhibition of melanin production by NMEB derivatives was attributable to B16F10 cTYR inhibitory activity or cytotoxicity to B16F10 cells, cell viability and B16F10 cTYR inhibitory activity of NMEB derivatives were examined.

### 2.9 Cell Viability

B16F10 cells were incubated for 72 h with NMEB derivatives **1a**–**1m** at 20, 10, and 5 μM. Following treatment with EZ‐Cytox solution for 2 h, absorbance at 450 nm was obtained to determine cell viability.

Figure S3 shows that thirteen NMEB derivatives demonstrated no detectable cytotoxicity at any tested concentration, suggesting that inhibition of melanin production by NMEB derivatives in Figure 5 is not due to cytotoxicity.

### 2.10 cTYR Inhibitory Activity in B16F10 Cells

NMEB derivatives **1e**–**1k**, which most potently inhibited B16F10‐cell melanin production, were used to assess cTYR inhibition in B16F10 cells. After culturing for 24 h, B16F10 cells were exposed to NMEB derivatives, and after 1 h, the previously described melanin production stimulators were added at previously used concentrations. cTYR activity was assessed by measuring absorbance at 475 nm after 72 h of incubation. NMEB inhibitory activity was benchmarked against 20 μM KA (positive control). Derivatives were assessed at 5, 10, and 20 μM.

**FIGURE 6:**
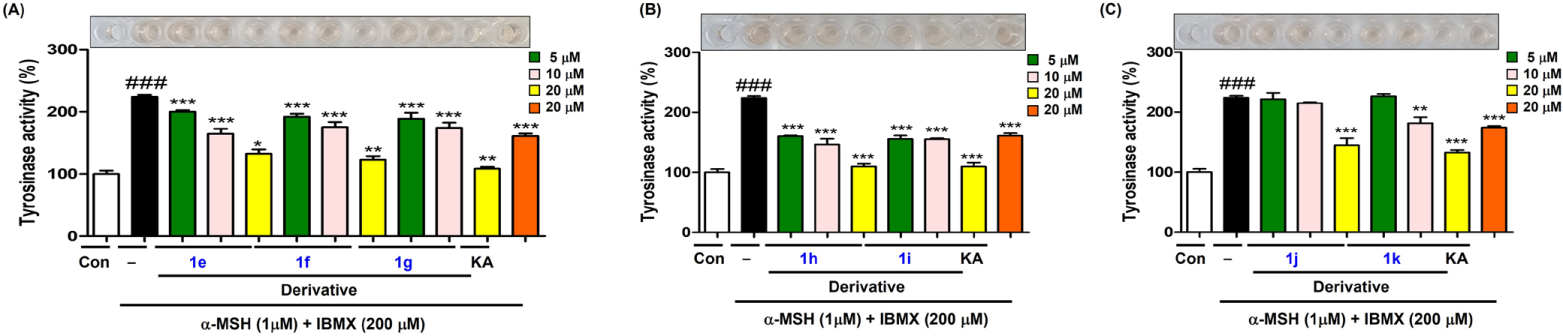
Impact of derivatives **1e**–**1k** (5, 10, and 20 μM) on B16F10 cTYR activity. The B16F10 cTYR activity of derivatives 1e, 1f, and 1g; 1h and 1i; and 1j and 1k are shown in (A), (B), and (C), respectively. Cells were incubated with test samples 1 h before the addition of stimulators (200 μM IBMX plus 1 μM α‐MSH) and cultivated for 72 h. KA (20 μM) was used to benchmark activity. *p < 0.05, **p < 0.01, and ***p < 0.001 vs. group treated with stimulator; ^###^ *p* < 0.001 vs. control group.

Stimulator treatment elevated cTYR activity 2.2‐fold, and exposure to KA significantly inhibited cTYR activity (Figure 6 and Figure S63). Each of the seven tested derivatives exhibited concentration‐dependent inhibition and, at 20 μM, inhibited cTYR activity more potently than KA. NMEB‐mediated cTYR activity inhibition potencies resembled those against melanin production in B16F10 cells, implying that their inhibition of melanin production could be attributed to their cTYR inhibitory efficacy. Despite their lack of Cu(II)‐chelating capacity, NMEB derivatives strongly inhibited B16F10 cTYR activity. NMEB derivatives' TYR inhibitory activities are predicted to result from strong interactions with TYR amino acids. NMEB derivatives exhibited weak to moderate inhibitory activity against mTYR but potent inhibition of B16F10 cTYR. Similar results have been reported for other compounds in the literature (Gaweł‐Bęben et al. 2023; Jung, Park, Jeong, Kim, et al. 2026).

### 2.11 In Situ cTYR Activity Inhibition in B16F10 Cells by NMEB Derivatives

To confirm the cTYR inhibitory activity of the NMEB derivatives, we assessed their in situ impact on cTYR activity as an alternative method. As in the cTYR activity experiment, B16F10 cells were treated with NMEB derivatives **1e**–**1k** (20 μM), and stimulators were administered 1 h later. Following a 72‐h incubation period, l‐DOPA was added to stimulate melanogenesis. After 2 h, melanin‐stained cells were photographed. cTYR activity was benchmarked to that of 20 μM KA.

As displayed in Figure S4, exposure to stimulators greatly increased the number of strongly melanin‐stained cells compared to the control (Figures S64–S73). Exposure to KA reduced the number of melanin‐stained cells induced by stimulators. Exposure to NMEB derivatives inhibited in situ cTYR activity more potently than KA treatment. These results were consistent for all NMEB derivatives tested (**1e**–**1k**). The results of in situ cTYR activity inhibition were very similar to the results of B16F10 cTYR inhibition shown in Figure 6.

### 2.12 Inhibition of Zebrafish Larvae Pigmentation

Although NMEB derivatives did not chelate Cu(II) ions, some derivatives showed slightly weaker or more potent mTYR inhibition than KA, and NMEB derivatives **1a**–**1m** all inhibited melanin biosynthesis in B16F10 cells more strongly than KA. These findings prompted us to investigate the ability of the derivatives to inhibit pigmentation in zebrafish larvae. Zebrafish larvae have been widely used as an animal model for screening and developing depigmenting agents (Ferreira et al. 2023; Rosa et al. 2022; Wu et al. 2023). We obtained preliminary zebrafish larval depigmentation results for derivatives **1a**–**1m** (0.1 mM) to guide selection of derivatives for more in‐depth in vivo zebrafish larval depigmentation experiments. Derivatives **1a** and **1e**, which most potently depigmented zebrafish larvae in the preliminary experiments, were selected for our in‐depth experiments. Zebrafish embryos were dechorionated at 24 h post‐fertilization (hpf). After 4 h, these dechorionated zebrafish embryos were exposed to derivatives **1a** and **1e** and KA. The derivatives were tested at 0.025 and 0.1 mM, and KA was tested at 20 mM, a concentration 200–800 times higher than the derivatives' test concentrations. Pigmentation was determined at 76 hpf by photographing lateral and dorsal views of zebrafish larvae.

**FIGURE 7:**
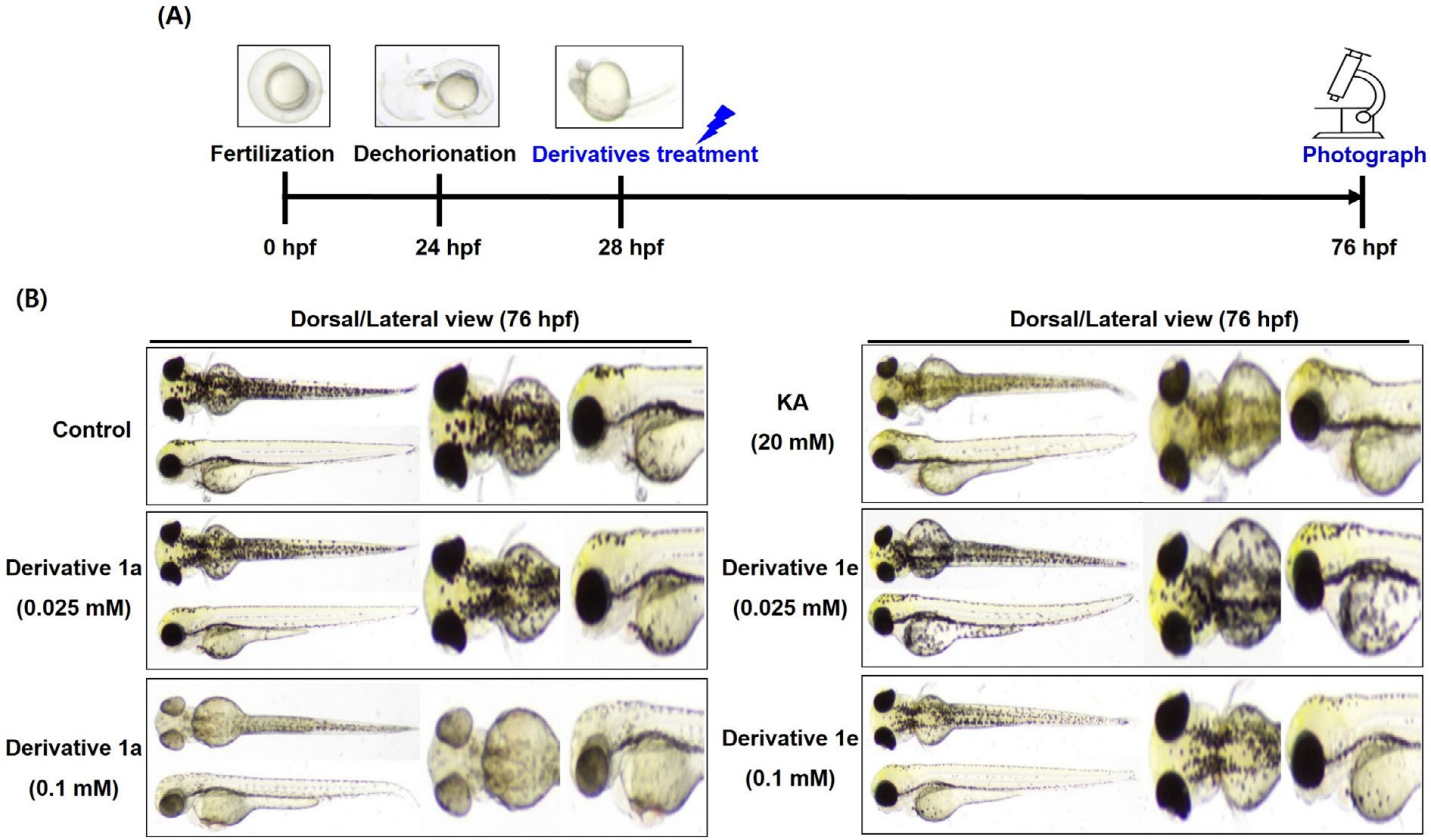
Inhibition of melanin formation by KA and NMEB derivatives **1a** and **1e** in zebrafish larvae. (A) Manipulation over time. (B) Photographs of zebrafish larvae exposed to KA (20 mM), **1a** (0.025 and 0.1 mM), and **1e** (0.025 and 0.1 mM) at 76 hpf (hours post‐fertilization). Zebrafish larvae were photographed from both dorsal and lateral views.

KA treatment weakly attenuated control group‐level pigmentation in zebrafish larvae (Figure 7 and Figures S74–S76). Derivatives **1a** and **1e** dose‐dependently inhibited zebrafish larval pigmentation. Derivative **1a** exhibited particularly potent depigmentation efficacy at 0.1 mM, a concentration much lower than that of KA. Zebrafish larvae eye pigmentation almost disappeared in the presence of the 0.1 mM derivative **1a**. Despite its limited mTYR inhibitory ability, **1e** effectively inhibited cTYR in B16F10 cells, thereby blocking melanin production. Derivative **1a** also demonstrated potent antimelanogenesis in B16F10 cells. Derivative **1a** also strongly inhibited pigmentation in zebrafish larvae in vivo. These disparate results between mTYR and other TYRs are probably attributable to differences in TYR structure and intracellular location. Mammalian TYRs are glycosylated transmembrane proteins located within melanosomes, whereas mTYRs are nonglycosylated tetramers located in the cytosol (Kwon et al. 1987; Pillaiyar et al. 2017). Derivative **1a** appeared to inhibit zebrafish larval pigmentation more effectively than other compounds reported in recent literature (Hsieh et al. 2025; Shi et al. 2026; Wang et al. 2025; Yang et al. 2025).

### 2.13 Cytotoxicity in HaCaT Cells

Keratinocytes are one of the primary skin cell types (Barker et al. 1991; Johansen 2017). We thus assessed the cytotoxic effects of NMEB derivatives on HaCaT (keratinocyte) cells. After incubating derivatives **1a**–**1m** with HaCaT cells for 24 h, cell viability results were obtained utilizing an EZ‐Cytox kit. NMEB derivatives were treated at 20, 10, and 5 μM.

The derivatives demonstrated no significant cytotoxicity at all tested concentrations (Figure S77), raising the possibility that NMEB derivatives may be used as cosmetics or to treat pigment‐related skin diseases.

### 2.14 Inhibition of Potato Juice Browning

Crop browning is also caused by TYR activity (Yu et al. 2022). NMEB derivatives were thus examined for their ability to suppress potato juice browning. NMEB derivatives **1a**–**1m** and KA (positive control) were each tested at 4 mM, and potato juice browning was quantitated after 24 h.

Progressive potato juice browning was observed in all test samples (Figure S78). Among the test samples, KA exerted the greatest preventative effect against potato juice browning. Among the NMEB derivatives, **1h** most potently suppressed potato juice browning. Its potency in preventing browning was similar to that of KA at 2 h after treatment but declined greatly relative to KA over time.

Considering the results of mTYR inhibitory activity and potato juice browning, NMEB derivatives appear to be more suitable for inhibiting mammalian and fish TYRs than fungal and plant‐derived TYRs.

### 2.15 In Silico ADMET Profiles and Drug‐Likeness of Selected NMEB Derivatives

To evaluate the drug‐likeness and pharmacokinetic feasibility of the representative NMEB derivatives **1k** and **1m**, an in silico ADMET prediction was performed. The comprehensive prediction results, including the detailed pharmacokinetic parameters and toxicity profiles, are provided in Table S1 (Table S1) and discussed in the Data S1 (Figure S80).

## 3 Conclusion

Thirteen *N*‐(2‐mercaptoethyl)benzamide (NMEB) derivatives, **1a**–**1m**, were synthesized as candidate TYR inhibitors. Derivative **1m** exhibited stronger inhibitory activity against mTYR than KA with l‐DOPA as a substrate. The other derivatives exhibited lower mTYR inhibitory activity than KA. All NMEB derivatives inhibited melanogenesis in B16F10 cells more potently than KA. These derivatives potently inhibited cTYR activity in B16F10 cells and similarly inhibited in situ B16F10 cTYR activity. The similar potencies of melanogenesis and cTYR inhibition in B16F10 cells suggest that the melanogenesis‐inhibiting effect of NMEB derivatives may be attributed to their capacity to inhibit cTYR activity. Derivative **1m** also effectively inhibited pigmentation in zebrafish larvae. These study results provide fundamental data that can contribute to the discovery of new TYR inhibitors. Many TYR inhibitors are being developed based on their mTYR inhibitory activity. However, compounds with low inhibitory activity against mTYR but strong inhibitory activity on mammalian and fish TYRs are constantly reported, as in this study. This study points out limitations in the development of novel TYR inhibitors based on mTYR inhibition results.

## 4 Experimental Section

All experimental procedures, including chemical synthesis, kinetic studies, cell culture assays, potato juice browning, in vivo zebrafish evaluations, and in silico ADMET predictions, are detailed in the Data S1.

## Author Contributions

**Minchang Kim:** resources, software, data curation. **Hee Jin Jung:** methodology, formal analysis, investigation, writing – original draft. **Yeonsoo Jeong:** investigation, software. **Hyunju Lee:** resources, investigation, validation. **Hae Young Chung:** writing – review and editing. **Hyejin Kang:** resources, investigation. **Hyeon Seo Park:** resources, methodology. **Hyung Ryong Moon:** conceptualization, supervision, funding acquisition, writing – original draft, writing – review and editing. **Hyunhee Ju:** software, resources, visualization.

## Funding

This work was supported by a 2‐Year Research Grant from Pusan National University.

## Conflicts of Interest

The authors declare no conflicts of interest.

## Supporting information


**Data S1:** cbdd70383‐sup‐0001‐Supinfo1.docx. **S1**–**S49:** ^1^H/^13^C NMR and HRMS spectra of **1a**–**m**, **2a**–**i**, and **2l**. **S50**–**S59:** Graphs used to calculate the IC_50_ values for derivatives **1f**–**m** and kojic acid. **S60:** Copper chelation capacity of **1a**–**m**. **S61:** Photo and original data for melanin production results of **1a**–**m** at 20 μM in B16F10 cells. **S62:** Melanin content levels in the presence of **1e**–**g** and **1h**–**k** at 5, 10, and 20 μM in B16F10 cells. **S63:** Impact of **1e**–**k** at 5, 10, and 20 μM on B16F10 cellular TYR activity. **S64**–**S73:** Images of the control group, α‐MSH + IBMX group, kojic acid group, and derivative (**1e**–**k**) group in the in situ B16F10 cellular TYR activity experiments. **S74**–**S76:** Melanin formation inhibitory activity results performed using zebrafish larvae. **S77:** Effect of **1a**–**m** on HaCaT cells. **S78:** Browning of potato juice in the presence of KA and **1a**–**m**. **S79:** Alignment of the redocked ligand and co‐crystallized ligand with the 2Y9X protein. **S80:** In silico ADMET profiles and drug‐likeness of **1k** and **1m**.
**Table S1:** Predicted ADMET profiles of NMEB derivatives **1k** and **1m** using ADMETlab 3.0.

## Data Availability

Data are available in the manuscript and Supporting Information—S1.
