# Supplementary material for: Tyrosinase Inhibitors With Enhanced Potency in Mammalian and Vertebrate Models, N‐(2‐Mercaptoethyl)benzamide Derivatives: Design, Synthesis, and Evaluation
Source: Chem Biol Drug Des. 2026 Aug 23;108(2):e70383. doi: 10.1111/cbdd.70383 (PMC13501074; doi:10.1111/cbdd.70383)
Supplement: Supplementary file 1 [file CBDD-108-e70383-s001.docx]

**Supporting Information**

**For**

**Tyrosinase Inhibitors with Enhanced Potency in Mammalian and Vertebrate Models, *N*-(2-Mercaptoethyl)benzamide Derivatives: Design, Synthesis, and Evaluation**

Hyunhee Ju ^a,1^, Minchang Kim ^a,1^, Hyunju Lee ^a,1^, Hee Jin Jung ^a,1^, Yeonsoo Jeong ^a^, Hyejin Kang ^a^, Hyeon Seo Park ^a^, Hae Young Chung ^b^, Hyung Ryong Moon ^a,^*

*^a^Department of Manufacturing Pharmacy, College of Pharmacy and Research Institute for Drug Development, Pusan National University, Busan 46241, Republic of Korea*

*^b^Department of Pharmacy, College of Pharmacy, Pusan National University, Busan 46241, Republic of Korea*

**Contents**

S1. ^1^H NMR spectrum of derivative **1a**	7

S2. ^13^C NMR spectrum of derivative **1a**	8

S3. ^1^H NMR spectrum of derivative **2a**	9

S4. ^13^C NMR spectrum of derivative **2a**	10

S5. ^1^H NMR spectrum of derivative **1b**	11

S6. ^13^C NMR spectrum of derivative **1b**	12

S7. HRMS spectrum of derivative **1b**	13

S8. ^1^H NMR spectrum of derivative **2b**	15

S9. ^1^H NMR spectrum of derivative **1c**	16

S10. ^13^C NMR spectrum of derivative **1c**	17

S11. HRMS spectrum of derivative **1c**	18

S12. ^1^H NMR spectrum of derivative **2c**	20

S13. ^13^C NMR spectrum of derivative **2c**	21

S14. ^1^H NMR spectrum of derivative **1d**	22

S15. ^13^C NMR spectrum of derivative **1d**	23

S16. ^1^H NMR spectrum of derivative **2d**	24

S17. ^13^C NMR spectrum of derivative **2d**	25

S18. ^1^H NMR spectrum of derivative **1e**	26

S19. ^13^C NMR spectrum of derivative **1e**	27

S20. HRMS spectrum of derivative **1e**	28

S21. ^1^H NMR spectrum of derivative **2e**	29

S22. ^13^C NMR spectrum of derivative **2e**	30

S23. ^1^H NMR spectrum of derivative **1f**	31

S24. ^13^C NMR spectrum of derivative **1f**	32

S25. HRMS spectrum of derivative **1f**	33

S26. ^1^H NMR spectrum of derivative **2f**	34

S27. ^13^C NMR spectrum of derivative **2f**	35

S28. ^1^H NMR spectrum of derivative **1g**	36

S29. ^13^C NMR spectrum of derivative **1g**	37

S30. HRMS spectrum of derivative **1g**	38

S31. ^1^H NMR spectrum of derivative **2g**	40

S32. ^13^C NMR spectrum of derivative **2g**	41

S33. ^1^H NMR spectrum of derivative **1h**	42

S34. ^13^C NMR spectrum of derivative **1h**	43

S35. HRMS spectrum of derivative **1h**	44

S36. ^1^H NMR spectrum of derivative **2h**	46

S37. ^1^H NMR spectrum of derivative **1i**	47

S38. ^13^C NMR spectrum of derivative **1i**	48

S39. ^1^H NMR spectrum of derivative **2i**	49

S40. ^1^H NMR spectrum of analog **1j**	50

S41. ^13^C NMR spectrum of derivative **1j**	51

S42. ^1^H NMR spectrum of derivative **1k**	52

S43. ^13^C NMR spectrum of derivative **1k**	53

S44. HRMS spectrum of derivative **1k**	54

S45. ^1^H NMR spectrum of derivative **1l**	56

S46. ^13^C NMR spectrum of derivative **1l**	57

S47. ^1^H NMR spectrum of derivative **2l**	58

S48. ^1^H NMR spectrum of derivative **1m**	59

S49. ^13^C NMR spectrum of derivative **1m**	60

S50. Graphs used to calculate the IC_50_ value for derivative **1f** and **1g** in the presence of l-tyrosine .................................................................................................................................... 61

S51. Graphs used to calculate the IC_50_ value for derivative **1h** and **1i** in the presence of l-tyrosine .................................................................................................................................... 62

S52. Graphs used to calculate the IC_50_ value for derivative **1j** and **1k** in the presence of l-tyrosine .................................................................................................................................... 63

S53. Graphs used to calculate the IC_50_ value for derivative **1l** and **1m** in the presence of l-tyrosine .................................................................................................................................... 64

S54. Graphs used to calculate the IC_50_ value for kojic acid in the presence of l-tyrosine .................................................................................................................................... 65

S55. Graphs used to calculate the IC_50_ value for derivative **1f** and **1g** in the presence of l-dopa ......................................................................................................................................... 66

S56. Graphs used to calculate the IC_50_ value for derivative **1h** and **1i** in the presence of l-dopa ......................................................................................................................................... 67

S57. Graphs used to calculate the IC_50_ value for derivative **1j** and **1k** in the presence of l-dopa ......................................................................................................................................... 68

S58. Graphs used to calculate the IC_50_ value for derivative **1l** and **1m** in the presence of l-dopa ......................................................................................................................................... 69

S59. Graphs used to calculate the IC_50_ value for kojic acid in the presence of l-dopa ......................................................................................................................................... 70

S60. Copper chelation capacity of NMEB derivatives **1a**–**1m** ............................................... 71

S61. Photo (A) and original data (B) for melanin production results of **1a**–**1m** at 20 μM in B16F10 cells ........................................................................................................................... 72

S62. Melanin content levels in the presence of NMEB derivatives **1e**–**1g** (A), **1h** and **1i** (B), and **1j** and **1k** (C) at three different concentrations (5, 10, and 20 μM) in B16F10 cells........ 73

S63. Impact of derivatives of derivatives **1e**–**1g** (A), **1h** and **1i** (B), and **1j** and **1k** (C) at three different concentrations (5, 10, and 20 μM) on B16F10 cellular tyrosinase activity………... 74

S64. Images of the control group (*n* = 9) in the in situ B16F10 cellular tyrosinase activity experiments ............................................................................................................................. 75

S65. Images of the α-MSH + IBMX group (*n* = 9) in the in situ B16F10 cellular tyrosinase activity experiments ................................................................................................................ 76

S66. Images of the kojic acid (20 μM) group (*n* = 8) in the in situ B16F10 cellular tyrosinase activity experiments ................................................................................................................ 77

S67. Images of the derivative **1e** (20 μM) group (*n* = 8) in the in situ B16F10 cellular tyrosinase activity experiments ................................................................................................................ 78

S68. Images of the derivative **1f** (20 μM) group (*n* = 9) in the in situ B16F10 cellular tyrosinase activity experiments ................................................................................................................ 79

S69. Images of the the derivative **1g** (20 μM) group (*n* = 8) in the in situ B16F10 cellular tyrosinase activity experiments ............................................................................................... 80

S70. Images of the derivative **1h** (20 μM) group (*n* = 8) in the in situ B16F10 cellular tyrosinase activity experiments ................................................................................................................ 81

S71. Images of the derivative **1i** (20 μM) group (*n* = 9) in the in situ B16F10 cellular tyrosinase activity experiments ................................................................................................................ 82

S72. Images of the derivative **1j** (20 μM) group (*n* = 8) in the in situ B16F10 cellular tyrosinase activity experiments ................................................................................................................ 83

S73. Images of the derivative **1k** (20 μM) group (*n* = 8) in the in situ B16F10 cellular tyrosinase activity experiments ................................................................................................................ 84

S74. Melanin formation inhibitory activity results performed using zebrafish larvae…......... 85

S75. Melanin formation inhibitory activity results performed using zebrafish larvae............ 86

S76. Melanin formation inhibitory activity results performed using zebrafish larvae............ 87

S77. Effect of derivatives **1a**–**1m** on HaCaT cells ................................................................... 88

S78. Browning of potato juice in the presence of KA and NMEB derivatives **1a**–**1m**……….89

S79. Alignment of the redocked ligand (green) and co-crystallized ligand (red) with the 2Y9X protein ..................................................................................................................................... 90

S80. In silico ADMET profiles and drug-likeness of selected NMEB derivatives.................. ..91

**STable 1.** Predicted ADMET profiles of NMEB derivatives **1k** and **1m** using ADMETlab 3.0............................................................................................................................................ 92

**SFigure 1.** Dixon plots of derivatives **1h** (A), **1k** (B), and **1m** (C) obtained from kinetic experiments.............................................................................................................................. 93

**SFigure 2.** mTYR allosteric site docking results. .................................................................... 94

**SFigure 3.** Cytotoxic effect of derivatives **1a**–**1m** on B16F10 cells........................................ 95

**SFigure 4.** Results of *in situ* B16F10 cTYR activity inhibition with NMEB derivatives **1e**–**1k** and KA..................................................................................................................................... 96

**1. Experimental Section**......................................................................................................... 97

**References**.............................................................................................................................110

S1. ^1^H NMR spectrum of derivative **1a**

S2. ^13^C NMR spectrum of derivative **1a**

S3. ^1^H NMR spectrum of derivative **2a**

S4. ^13^C NMR spectrum of derivative **2a**

S5. ^1^H NMR spectrum of derivative **1b**

S6. ^13^C NMR spectrum of derivative **1b**

S7. HRMS spectrum of derivative **1b**

S7. HRMS spectrum of derivative **1b** (continued)

S8. ^1^H NMR spectrum of derivative **2b**

S9. ^1^H NMR spectrum of derivative **1c**

S10. ^13^C NMR spectrum of derivative **1c**

S11. HRMS spectrum of derivative **1c**

S11. HRMS spectrum of derivative **1c** (continued)

S12. ^1^H NMR spectrum of derivative **2c**

S13. ^13^C NMR spectrum of derivative **2c**

S14. ^1^H NMR spectrum of derivative **1d**

S15. ^13^C NMR spectrum of derivative **1d**

S16. ^1^H NMR spectrum of derivative **2d**

S17. ^13^C NMR spectrum of derivative **2d**

S18. ^1^H NMR spectrum of derivative **1e**

S19. ^13^C NMR spectrum of derivative **1e**

S20. HRMS spectrum of derivative **1e**

S21. ^1^H NMR spectrum of derivative **2e**

S22. ^13^C NMR spectrum of derivative **2e**

S23. ^1^H NMR spectrum of derivative **1f**

S24. ^13^C NMR spectrum of derivative **1f**

S25. HRMS spectrum of derivative **1f**

S26. ^1^H NMR spectrum of derivative **2f**

S27. ^13^C NMR spectrum of derivative **2f**

S28. ^1^H NMR spectrum of derivative **1g**

S29. ^13^C NMR spectrum of derivative **1g**

S30. HRMS spectrum of derivative **1g**

S30. HRMS spectrum of derivative **1g** (continued)

S31. ^1^H NMR spectrum of derivative **2g**

S32. ^13^C NMR spectrum of derivative **2g**

 S33. ^1^H NMR spectrum of derivative **1h**

 S34. ^13^C NMR spectrum of derivative **1h**

S35. HRMS spectrum of derivative **1h**

S35. HRMS spectrum of derivative **1h** (continued)

S36. ^1^H NMR spectrum of derivative **2h**

S37. ^1^H NMR spectrum of derivative **1i**

S38. ^13^C NMR spectrum of derivative **1i**S39. ^1^H NMR spectrum of derivative **2i**

S40. ^1^H NMR spectrum of derivative **1j**

S41. ^13^C NMR spectrum of derivative **1j**

S42. ^1^H NMR spectrum of derivative **1k**

S43. ^13^C NMR spectrum of derivative **1k**

S44. HRMS spectrum of derivative **1k**

S44. HRMS spectrum of derivative **1k** (continued)

S45. ^1^H NMR spectrum of derivative **1l**

S46. ^13^C NMR spectrum of derivative **1l**

S47. ^1^H NMR spectrum of derivative **2l**

S48. ^1^H NMR spectrum of analog **1m**

S49. ^13^C NMR spectrum of derivative **1m**

S50. Graphs used to calculate the IC_50_ value for derivative **1f** and **1g** in the presence of l-tyrosine

S51. Graphs used to calculate the IC_50_ value for derivative **1h** and **1i** in the presence of l-tyrosine

S52. Graphs used to calculate the IC_50_ value for derivative **1j** and **1k** in the presence of l-tyrosine

S53. Graphs used to calculate the IC_50_ value for derivative **1l** and **1m** in the presence of l-tyrosine

S54. Graphs used to calculate the IC_50_ value for kojic acid in the presence of l-tyrosine

S55. Graphs used to calculate the IC_50_ value for derivative **1f** and **1g** in the presence of l-dopa

S56. Graphs used to calculate the IC_50_ value for derivative **1h** and **1i** in the presence of l-dopa

S57. Graphs used to calculate the IC_50_ value for derivative **1j** and **1k** in the presence of l-dopa

S58. Graphs used to calculate the IC_50_ value for derivative **1l** and **1m** in the presence of l-dopa

S59. Graphs used to calculate the IC_50_ value for kojic acid in the presence of l-dopa

S60. Copper chelation capacity of NMEB derivatives **1a**–**1m**

S61. Photo (A) and original data (B) for melanin production results of **1a**–**1m** at 20 μM in B16F10 cells

S62. Melanin content levels in the presence of NMEB derivatives **1e**–**1g** (A), **1h** and **1i** (B), and **1j** and **1k** (C) at three different concentrations (5, 10, and 20 μM) in B16F10 cells. Melanin inhibition effect was compared using kojic acid (KA: 20 μM)

S63. Impact of derivatives of derivatives **1e**–**1g** (A), **1h** and **1i** (B), and **1j** and **1k** (C) at three different concentrations (5, 10, and 20 μM) on B16F10 cellular tyrosinase activity. Their inhibitory effect on cellular tyrosinase activity was compared using kojic acid (KA: 20 μM)

S64. Images of the control group (*n* = 9) in the in situ B16F10 cellular tyrosinase activity experiments

S65. Images of the α-MSH + IBMX group (*n* = 9) in the in situ B16F10 cellular tyrosinase activity experiments

S66. Images of the kojic acid (20 μM) group (*n* = 8) in the in situ B16F10 cellular tyrosinase activity experiments

S67. Images of the derivative **1e** (20 μM) group (*n* = 8) in the in situ B16F10 cellular tyrosinase activity experiments

S68. Images of the derivative **1f** (20 μM) group (*n* = 9) in the in situ B16F10 cellular tyrosinase activity experiments

S69. Images of the the derivative **1g** (20 μM) group (*n* = 8) in the in situ B16F10 cellular tyrosinase activity experiments

S70. Images of the derivative **1h** (20 μM) group (*n* = 8) in the in situ B16F10 cellular tyrosinase activity experiments

S71. Images of the derivative **1i** (20 μM) group (*n* = 9) in the in situ B16F10 cellular tyrosinase activity experiments

S72. Images of the derivative **1j** (20 μM) group (*n* = 8) in the in situ B16F10 cellular tyrosinase activity experiments

S73. Images of the derivative **1k** (20 μM) group (*n* = 8) in the in situ B16F10 cellular tyrosinase activity experiments

S74. Melanin formation inhibitory activity results performed using zebrafish larvae. Dorsal and lateral views of zebrafish larvae treated with control (A) and KA (kojic acid, 20 mM)

S75. Melanin formation inhibitory activity results performed using zebrafish larvae. Dorsal and lateral views of zebrafish larvae treated with derivative **1a** (0.025 mM, A) and **1a** (0.1 mM, B)

S76. Melanin formation inhibitory activity results performed using zebrafish larvae. Dorsal and lateral views of zebrafish larvae treated with derivative **1e** (0.025 mM, A) and **1e** (0.1 mM, B)

S77. Effect of derivatives **1a**–**1m** on HaCaT cells

S78. Browning of potato juice in the presence of KA and NMEB derivatives **1a**–**1m**. All samples were tested at 4 mM, and browning of potato juice was photographed at 0, 1, 2, 3, 4, 5, 6, 7, and 24 h.

S79. Alignment of the redocked ligand (green) and co-crystallized ligand (red) with the 2Y9X protein.

The ligand used is tropolone. The validation of the docking procedure was carried out by redocking the co-crystallized tropolone into the tyrosinase active site. In the redocking results, it was found that the redocked tropolone reproduced the binding pose with a binding affinity of −5.5 kcal/mol. The RMSD of the co-crystallized and experimental poses was analyzed, and the RMSD value was 0.61 Å. These results suggest that the docking simulation could properly accommodate the crystallized ligand.

S80. In silico ADMET profiles and drug-likeness of selected NMEB derivatives

The in silico ADMET profiles of the representative NMEB derivatives **1k** and **1m** were evaluated using ADMETlab 3.0 to assess their drug-likeness and pharmacokinetic feasibility. Both derivatives strictly adhered to the Lipinski and GSK rules, indicating favorable medicinal chemistry properties for drug development (STable 1). Notably, both compounds exhibited exceptionally low predicted probabilities (≤ 0.047) for cytotoxicity against A549 and Hek293 cell lines, suggesting an excellent cellular safety profile. In terms of mutagenicity, the derivatives displayed moderate AMES toxicity probabilities ranging from 0.558 to 0.664. While they were identified as CYP1A2 inhibitors rather than substrates, both compounds demonstrated favorable excretion profiles with stable plasma clearance rates and reasonable half-lives (T1/2 ≤ 1.195).

**STable 1.** Predicted ADMET profiles of NMEB derivatives **1k** and **1m** using ADMETlab 3.0

| **Physicochemical property** | | **Physicochemical property** | |
| --- | --- | --- | --- |
| logS | -3.687 | logS | -1.947 |
| logP | 3.089 | logP | 1.482 |
| pka | 7.854 | pka | 9.224 |
| **Medicinal chemistry** | | **Medicinal chemistry** | |
| Lipinski rule | Accepted | Lipinski rule | Accepted |
| GSK rule | Accepted | GSK rule | Accepted |
| **Absorption** | | **Absorption** | |
| Caco-2 permeability | -4.858 | Caco-2 permeability | -5.177 |
| Pgp substrate | --- | Pgp substrate | --- |
| **Distribution** | | **Distribution** | |
| Plasma protein binding | 91.8% | Plasma protein binding | 62.6% |
| **Metabolism** | | **Metabolism** | |
| CYP1A2 inhibitor | +++ | CYP1A2 inhibitor | +++ |
| CYP1A2 substrate | --- | CYP1A2 substrate | --- |
| CYP3A4 inhibitor | --- | CYP3A4 inhibitor | --- |
| CYP3A4 substrate | --- | CYP3A4 substrate | --- |
| **Excretion** | | **Excretion** | |
| CL_plasma_ | 8.946 | CL_plasma_ | 7.958 |
| T_1/2_ | 0.77 | T_1/2_ | 1.195 |
| **Toxicity** | | **Toxicity** | |
| hERG blockers | 0.038 | hERG blockers | 0.017 |
| AMES toxicity | 0.558 | AMES toxicity | 0.664 |
| A549 cytotoxicity | 0.047 | A549 cytotoxicity | 0.025 |
| Hek293 cytotoxicity | 0.045 | Hek293 cytotoxicity | 0.017 |

**SFigure 1.** Dixon plots of derivatives **1h** (A), **1k** (B), and **1m** (C) obtained from kinetic experiments. Four l-DOPA concentrations (8, 4, 2, and 1 mM) were assayed in the presence of each of four NMEB derivative concentrations (0, 12.5, 25, and 50 μM for **1h** and **1k** and 0, 5, 10, and 20 μM for **1m**).

**SFigure 2.** mTYR allosteric site docking results. Images of mTYR in complex with NMEB derivatives **1k** (yellow) and **1m** (red) are represented with mTYR in rounded ribbon (A) and space-filling (B) forms. Docking results (C) for NMEB derivative **1k** and docking results (D) for NMEB derivative **1m**. Green arrows, yellow waves, and red arrows respectively represent H-bonds with ligand as donor, HP interactions, and H-bonds with ligand as acceptor.

**SFigure 3.** Cytotoxic effect of derivatives **1a**–**1m** on B16F10 cells. Cells were incubated with **1a**–**1m** (20, 10, and 5 μM) for 72 h, and cell viability was determined using EZ-Cytox solution.

**SFigure 4.** Results of *in situ* B16F10 cTYR activity inhibition with NMEB derivatives **1e**–**1k** and KA. B16F10 cells were exposed to stimulators (IBMX and α-MSH) for 72 h following 1 h of sample treatment. After 2 h of l-DOPA treatment, melanin‑stained cells were observed. All test samples were treated at 20 μM. The red arrows indicate melanin-stained cells.

***1. Experimental Section***

*1.1. General methods in synthesis*

Confirmation of all prepared compound structures was performed using ^1^H and ^13^C NMR. The structures of some final compounds were further confirmed by comparing their NMR data to published data. CDCl_3_ and (CD_3_)_2_SO (DMSO-*d*_6_) were used as NMR solvents. 400 MHz ^1^H NMR and 100 MHz ^13^C NMR data were obtained utilizing a JEOL ECZ400S instrument (JEOL Ltd., Tokyo, Japan). Dichloromethane (DCM), which served as reaction solvent, was distilled over CaH_2_ to render it anhydrous. NMR data were recorded in ppm for chemical shifts and in Hz for coupling constants. Peak splittings were displayed as t (triplet), d (doublet), dddd (doublet of doublets of doublets of doublets), ddd (doublet of doublets of doublets), dd (doublet of doublets), pq (pseudoquartet), brt (broad triplet), m (multiplet), s (singlet), td (triplet of doublets), and dt (doublet of triplets). Data for high-resolution (HR) mass spectroscopy (MS) data were gathered in the ESI(+)-QTOF mode with an HR liquid chromatography tandem mass spectrometer (Sciex's ZenoTOF 7600 model; Toronto, ON, Canada).

*1.1.1. General synthetic procedures for* ***1a****–****1m****,* ***2a****–****2i****, and* ***2l***

Cysteamine hydrochloride (80 mg, 0.70 mmol) and benzoyl chloride (0.67 equiv.) or an appropriately substituted benzoyl chloride (0.67 equiv.; 4-methylbenzoyl chloride, 3-methylbenzoyl chloride, 2-methylbenzoyl chloride, 3-fluorobenzoyl chloride, 3-chlorobenzoyl chloride, 3-bromobenzoyl chloride, 4-fluorobenzoyl chloride, 4-chlorobenzoyl chloride, 4-bromobenzoyl chloride, 3,4-dichlorobenzoyl chloride, 3,4-dimethoxybenzoyl chloride, or 3,4,5-trimethoxybenzoyl chloride) in DCM (4 mL) were stirred at 15–17 °C with trimethylamine (2.5 equiv.) for 5–10 h. After partitioning between an aqueous NaHCO_3_ solution and DCM, the DCM layer was concentrated under reduced pressure. For **1j**, **1k**, and **1m**, the residue was dissolved in methanol (4 mL), and the solution was stirred at 15–17 °C in the presence of K_2_CO_3_ (0.4–1.0 equiv.) for 7–12 h. After removing volatiles, the residue was purified via column chromatography (MeOH/DCM = 1:40–60; silica gel) to produce **1j**, **1k**, and **1m** in 68–81% yield. For **1a**–**1i**, **1l**, **2a**–**2i**, and **2l**, the residue was purified via column chromatography (MeOH/DCM = 1:40–60; silica gel) to produce benzamide derivatives **1a**–**1i** and **1l** and benzothioate derivatives **2a**–**2i** and **2l** in 35–54% and 24–42% yields, respectively. Similar to the syntheses of **1j**, **1k**, and **1m**, the obtained benzothioate derivatives **2a**–**2i** and **2l** were dissolved in methanol (2–3 mL), and the solution was stirred at 15–17 °C in the presence of K_2_CO_3_ (0.4–1.0 equiv.) for 5–10 h. After removing volatiles, the residue was purified by silica gel column chromatography (methanol/DCM = 1:40–60) to produce **1a**–**1i** and **1l** in 79–94% yield.

^1^H and ^13^C NMR spectra were obtained at 400 and 100 MHz, respectively. Proton peaks from thiol (SH) groups in the derivatives were not detected in the NMR spectra. Thiol proton peaks can be attenuated by fast hydrogen exchange (RSH_a_ + RSH_b_ RSH_b_ + RSH_a_), by the presence of water or acidic/basic impurities, or by temperature, all of which reduce its observability in NMR spectra (Jung et al., 2024; Li, Yang, Xiong, & Wang, 2021; Sirgamalla et al., 2018).

*1.1.2. Spectroscopic data for* ***1a****–****1m****,* ***2a****–****2i****, and* ***2l***

1.1.2.1. *S*-(2-Benzamidoethyl) benzothioate (**2a**) (Kollar et al. 2023)

^1^H NMR (CDCl_3_, 400 MHz) *δ* 7.97 (d, 2H, *J* = 7.2 Hz, Ph), 7.78 (d, 2H, *J* = 7.2 Hz, Ph), 7.59 (t, 1H, *J* = 7.2 Hz, 7.53–7.41 (m, 5H, Ph), 6.75 (brt, 1H, *J* = 5.6 Hz, NH), 3.77 (pq, 2H, *J* = 6.0, 5.6 Hz, NCH_2_), 3.37 (t, 2H, *J* = 6.0 Hz, SCH_2_); ^13^C NMR (CDCl_3_, 100 MHz) *δ* 192.9, 167.7, 136.8, 134.3, 133.9, 131.6, 128.8, 128.7, 127.4, 127.0, 40.6, 28.6.

1.1.2.2. *S*-(2-(2-Methylbenzamido)ethyl) 2-methylbenzothioate (**2b**)

^1^H NMR (CDCl_3_, 400 MHz) *δ* 7.80 (d, 1H, *J* = 7.6 Hz, Ph), 7.40 (t, 1H, *J* = 7.6 Hz, Ph), 7.36 (d, 1H, *J* = 7.6 Hz, Ph), 7.30 (t, 1H, *J* = 7.6 Hz, Ph), 7.26–7.16 (m, 4H, Ph), 6.21 (brt, 1H, *J* = 5.6 Hz, NH), 3.74 (pq, 2H, *J* = 6.4, 5.6 Hz, NCH_2_), 3.32 (t, 2H, *J* = 6.4 Hz, SCH_2_), 2.45 (s, 3H, Me), 2.43 (s, 3H, Me).

1.1.2.3. *S*-(2-(3-Methylbenzamido)ethyl) 3-methylbenzothioate (**2c**)

^1^H NMR (DMSO-*d*_6_, 400 MHz) *δ* 8.67 (t, 1H, *J* = 5.6 Hz, NH), 7.73–7.72 (m, 2H, Ph), 7.65–7.61 (m, 2H, Ph), 7.50 (d, 1H, *J* = 8.0 Hz, Ph), 7.45 (t, 1H, *J* = 8.0 Hz, Ph), 7.34–7.33 (m, 2H, Ph), 3.51 (pq, 2H, *J* = 6.8, 5.6 Hz, NCH_2_), 3.26 (t, 1H, *J* = 6.8 Hz, SCH_2_), 2.38 (s, 3H, Me), 2.34 (s, 3H, Me); ^13^C NMR (DMSO-*d*_6_, 100 MHz) *δ* 191.6, 167.0, 139.2, 138.1, 137.0, 135.1, 134.8, 132.3, 129.5, 128.7, 128.3, 127.6, 124.9, 124.6, 39.3, 28.8, 21.5, 21.3.

1.1.2.4. *S*-(2-(4-Methylbenzamido)ethyl) 4-methylbenzothioate (**2d**)

^1^H NMR (DMSO-*d*_6_, 400 MHz) *δ* 8.64 (t, 1H, *J* = 5.6 Hz, NH), 7.82 (d, 2H, *J* = 8.0 Hz, Ph), 7.74 (d, 2H, *J* = 8.0 Hz, Ph), 7.35 (d, 2H, *J* = 8.0 Hz, Ph), 7.26 (d, 2H, *J* = 8.0 Hz, Ph), 3.50 (pq, 2H, *J* = 6.8, 5.6 Hz, NCH_2_), 3.24 (t, 2H, *J* = 6.8 Hz, SCH_2_), 2.38 (s, 3H, Me), 2.34 (s, 3H, Me); ^13^C NMR (DMSO-*d*_6_, 100 MHz) *δ* 191.0, 166.7, 145.0, 141.6, 134.4, 132.0, 130.1, 129.3, 127.7, 127.4, 39.3, 28.7, 21.7, 21.5.

1.1.2.5. *S*-(2-(3-Fluorobenzamido)ethyl) 3-fluorobenzothioate (**2e**)

^1^H NMR (CDCl_3_, 400 MHz) *δ* 7.79–7.76 (m, 1H, Ph), 7.66–7.63 (m, 1H, Ph), 7.53–7.48 (m, 2H, Ph), 7.45 (td, 1H, *J* = 8.0, 5.2 Hz, Ph), 7.42–7.37 (m, 1H, Ph), 7.32–7.27 (m, 1H, Ph), 7.21–7.16 (m, 1H, Ph), 6.73 (brt, 1H, *J* = 5.6 Hz, NH), 3.76 (pq, 2H, *J* = 6.4, 5.6 Hz, NCH_2_), 3.37 (t, 2H, *J* = 6.4 Hz, SCH_2_); ^13^C NMR (CDCl_3_, 100 MHz) 191.8 (d, *J* = 2.7 Hz), 166.4 (d, *J* = 2.4 Hz), 162.8 (d, *J* = 246.3 Hz), 162.8 (d, *J* = 247.5 Hz), 138.6 (d, *J* = 2.7 Hz), 136.4, 130.5 (d, *J* = 6.9 Hz), 130.3 (d, *J* = 8.1 Hz), 123.3 (d, *J* = 3.9 Hz), 122.4 (d, *J* = 3.1 Hz), 120.9 (d, *J* = 19.3 Hz), 118.7 (d, *J* = 21.3 Hz), 114.5 (d, *J* = 23.5 Hz), 114.3 (d, *J* = 23.2 Hz), 40.6, 28.7.

1.1.2.6. *S*-(2-(3-Chlorobenzamido)ethyl) 3-chlorobenzothioate (**2f**)

^1^H NMR (DMSO-*d*_6_, 400 MHz) *δ* 8.86 (t, 1H, *J* = 5.6 Hz, NH), 7.90–7.85 (m, 3H, Ph), 7.81–7.75 (m, 2H, Ph), 7.61–7.57 (m, 2H, Ph), 7.50 (t, 1H, *J* = 8.0 Hz, Ph), 3.54 (pq, 2H, *J* = 6.4, 5.6 Hz, NCH_2_), 3.29 (t, 2H, *J* = 6.4 Hz, SCH_2_); ^13^C NMR (DMSO-*d*_6_, 100 MHz) *δ* 190.5, 165.5, 138.6, 136.7, 134.5, 134.2, 133.7, 131.7, 131.7, 130.9, 127.5, 126.7, 126.5, 126.2, 39.2, 29.0.

1.1.2.7. *S*-(2-(3-Bromobenzamido)ethyl) 3-bromobenzothioate (**2g**)

^1^H NMR (DMSO-*d*_6_, 400 MHz) *δ* 8.85 (t, 1H, *J* = 5.6 Hz, NH), 8.00 (t, 1H, *J* = 1.6 Hz, Ph), 7.98 (t, 1H, *J* = 1.6 Hz, Ph), 7.94–7.87 (m, 2H, Ph), 7.83 (dt, 1H, *J* = 8.0, 1.2 Hz, Ph), 7.74–7.72 (m, 1H, Ph), 7.52 (t, 1H, *J* = 8.0 Hz, Ph), 7.44 (t, 1H, *J* = 8.0 Hz, Ph), 3.53 (pq, 2H, *J* = 6.4, 5.6 Hz, NCH_2_), 3.29 (t, 2H, *J* = 6.4 Hz, SCH_2_); ^13^C NMR (CDCl_3_, 100 MHz) *δ* 190.5, 165.4, 138.8, 137.1, 136.9, 134.5, 131.9, 131.1, 130.4, 129.6, 126.9, 126.6, 122.8, 122.2, 39.2, 29.0.

1.1.2.8. *S*-(2-(4-Fluorobenzamido)ethyl) 4-fluorobenzothioate (**2h**) (Commercially available)

^1^H NMR (CDCl_3_, 400 MHz) *δ* 7.99 (dd, 2H, *J* = 8.8, 5.6 Hz, Ph), 7.78 (dd, 2H, *J* = 8.8, 5.6 Hz, Ph), 7.14 (t, 2H, *J* = 8.8 Hz, Ph), 7.09 (t, 2H, *J* = 8.8 Hz, Ph), 6.72 (brt, 1H, *J* = 5.6 Hz, NH), 3.75 (pq, 2H, *J* = 6.4, 5.6 Hz, NCH_2_), 3.35 (t, 2H, *J* = 6.4 Hz, SCH_2_).

1.1.2.9. *S*-(2-(4-Chlorobenzamido)ethyl) 4-chlorobenzothioate (**2i**) (Matthies et al. 1990)

^1^H NMR (CDCl_3_, 400 MHz) *δ* 7.90 (d, 2H, *J* = 8.8 Hz, Ph), 7.71 (d, 2H, *J* = 8.8 Hz, Ph), 7.44 (d, 2H, *J* = 8.8 Hz, Ph), 7.39 (d, 2H, *J* = 8.8 Hz, Ph), 6.72 (brt, 1H, *J* = 5.6 Hz, NH), 3.75 (pq, 2H, *J* = 6.4, 5.6 Hz, NCH_2_), 3.36 (t, 2H, *J* = 6.4 Hz, SCH_2_).

1.1.2.10. *S*-(2-(3,4-Dimethoxybenzamido)ethyl) 3,4-dimethoxybenzothioate (**2l**)

^1^H NMR (CDCl_3_, 400 MHz) *δ* 7.66 (dd, 1H, *J* = 8.4, 2.0 Hz, Ph), 7.45 (d, 1H, *J* = 2.0 Hz, Ph), 7.40 (d, 1H, *J* = 2.0 Hz, Ph), 7.31 (dd, 1H, *J* = 8.4, 2.0 Hz, Ph), 6.89 (d, 1H, *J* = 8.4 Hz, Ph), 6.85 (d, 1H, *J* = 8.4 Hz, Ph), 6.75 (brt, 1H, *J* = 5.2 Hz, NH), 3.94 (s, 3H, OMe), 3.92 (s, 3H, OMe), 3.91 (s, 3H, OMe), 3.90 (s, 3H, OMe), 3.74 (pq, 2H, *J* = 6.4, 5.2 Hz, NCH_2_), 3.34 (t, 2H, *J* = 6.4 Hz, SCH_2_).

1.1.2.11. *N*-(2-Mercaptoethyl)benzamide (**1a**) (Marichev et al., 2017; Petersson, Jenkins, & Loughlin, 2009)

^1^H NMR (DMSO-*d*_6_, 400 MHz) *δ* 8.65 (t, 1H, *J* = 5.6 Hz, NH), 7.84 (d, 2H, *J* = 7.6 Hz, Ph), 7.52 (t, 1H, *J* = 7.6 Hz, Ph), 7.46 (t, 2H, *J* = 7.6 Hz, Ph), 3.57 (pq, 2H, *J* = 6.8, 5.6 Hz, NCH_2_), 2.94 (t, 2H, *J* = 6.8 Hz, SCH_2_); ^13^C NMR (DMSO-*d*_6_, 100 MHz) *δ* 166.9, 134.8, 131.8, 128.8, 127.7, 39.3, 37.7.

1.1.2.12. *N*-(2-Mercaptoethyl)-2-methylbenzamide (**1b**)

^1^H NMR (DMSO-*d*_6_, 400 MHz) *δ* 8.38 (t, 1H, *J* = 5.6 Hz, NH), 7.34–7.29 (m, 2H, Ph), 7.24–7.19 (m, 2H, Ph), 3.53 (pq, 2H, *J* = 6.8, 5.6 Hz, NCH_2_), 2.94 (t, 2H, *J* = 6.8 Hz, SCH_2_), 2.33 (s, 3H, Me); ^13^C NMR (DMSO-*d*_6_, 100 MHz) *δ* 169.7, 137.6, 135.7, 130.9, 129.8, 127.5, 126.0, 38.8, 37.8, 19.9; HRMS (ESI+) *m*/*z* C_10_H_12_NOS (M + H – H_2_)^+^ calcd 194.0640, obsd 164.0630.

1.1.2.13. *N*-(2-Mercaptoethyl)-3-methylbenzamide (**1c**)

^1^H NMR (CDCl_3_, 400 MHz) *δ* 7.59 (brs, 1H, Ph), 7.56–7.53 (m, 1H, Ph), 7.31–7.27 (m, 2H, Ph), 6.81 (brt, 1H, *J* = 5.6 Hz, NH), 3.78 (pq, 2H, *J* = 6.4, 5.6 Hz, NCH_2_), 3.34 (t, 2H, *J* = 6.4 Hz, SCH_2_), 2.40 (s, 3H, Me); ^13^C NMR (DMSO-*d*_6_, 100 MHz) *δ* 168.0, 138.5, 134.2, 132.4, 128.5, 127.9, 124.1, 39.2, 38.0, 21.4; HRMS (ESI+) *m*/*z* C_10_H_12_NOS (M + H – H_2_)^+^ calcd 194.0640, obsd 164.0634.

1.1.2.14. *N*-(2-Mercaptoethyl)-4-methylbenzamide (**1d**) (El-Gendy, Ghazvini Zadeh, Sotuyo, Pillai, & Katritzky, 2013)

^1^H NMR (DMSO-*d*_6_, 400 MHz) *δ* 8.57 (t, 1H, *J* = 5.6 Hz, NH), 7.74 (d, 2H, *J* = 8.0 Hz, Ph), 7.25 (d, 2H, *J* = 8.0 Hz, Ph), 3.54 (pq, 2H, *J* = 6.8, 5.6 Hz, NCH_2_), 2.92 (t, 2H, *J* = 6.8 Hz, SCH_2_), 2.34 (s, 3H, Me); ^13^C NMR (DMSO-*d*_6_, 100 MHz) *δ* 166.7, 141.6, 132.0, 129.3, 127.7, 39.3, 37.7, 21.5.

1.1.2.15. 3-Fluoro-*N*-(2-mercaptoethyl)benzamide (**1e**)

^1^H NMR (DMSO-*d*_6_, 400 MHz) *δ* 8.75 (t, 1H, *J* = 5.6 Hz, NH), 7.69 (dt, *J* = 8.0, 1.2 Hz, 2-H), 7.64–7.61 (m, 1H, 4-H), 7.52 (td, 1H, *J* = 8.0, 6.0 Hz, 5-H), 7.40–7.35 (m, 1H, 6-H), 3.56 (pq, 2H, *J* = 6.8, 5.6 Hz, NCH_2_), 2.94 (t, 2H, *J* = 6.8 Hz, SCH_2_); ^13^C NMR (DMSO-*d*_6_, 100 MHz) *δ* 165.5 (d, *J* = 2.7 Hz, C(=O)), 162.5 (d, *J* = 242.9 Hz, C3), 137.2 (d, *J* = 6.2 Hz), 131.0 (d, *J* = 7.9 Hz), 123.8 (d, *J* = 2.8 Hz), 118.7 (d, *J* = 21.0 Hz), 114.5 (d, *J* = 22.5 Hz), 39.3, 37.5; HRMS (ESI+) *m*/*z* C_9_H_9_FNOS (M + H – H_2_)^+^ calcd 198.0389, obsd 198.0380.

1.1.2.16*.* 3-Chloro-*N*-(2-mercaptoethyl)benzamide (**1f**)

^1^H NMR (DMSO-*d*_6_, 400 MHz) *δ* 8.78 (t, 1H, *J* = 6.0 Hz, NH), 7.88–7.79 (m, 2H, 2-H, 6-H), 7.61–7.58 (m, 1H, 4-H), 7.50 (t, 1H, *J* = 8.0 Hz, 5-H), 3.56 (pq, 2H, *J* = 6.8, 6.0 Hz, NCH_2_), 2.93 (t, 2H, *J* = 6.8 Hz, SCH_2_); ^13^C NMR (DMSO-*d*_6_, 100 MHz) *δ* 165.4, 136.8, 133.7, 131.6, 130.9, 127.5, 126.5, 39.3, 37.5; HRMS (ESI+) *m*/*z* C_9_H_9_ClNOS (M + H – H_2_)^+^ calcd 214.0093, obsd 214.0082.

1.1.2.17. 3-Bromo-*N*-(2-mercaptoethyl)benzamide (**1g**)

^1^H NMR (DMSO-*d*_6_, 400 MHz) *δ* 8.78 (t, 1H, *J* = 5.6 Hz, NH), 8.01 (pt, 1H, *J* = 2.0, 1.6 Hz, 2-H), 7.84 (ddd, 1H, *J* = 8.0, 1.6, 0.8 Hz, 6-H), 7.73 (ddd, 1H, *J* = 8.0, 2.0, 0.8 Hz, 4-H), 7.43 (t, 1H, *J* = 8.0 Hz, 5-H), 3.56 (pq, 2H, *J* = 6.8, 5.6 Hz, NCH_2_), 2.93 (t, 2H, *J* = 6.8 Hz, SCH_2_); ^13^C NMR (CDCl_3_, 100 MHz) *δ* 166.5, 136.3, 134.6, 130.4, 130.1, 125.7, 122.9, 39.5, 38.1; HRMS (ESI+) *m*/*z* C_9_H_11_BrNOS (M + H)^+^ calcd 259.9745, obsd 259.9730.

1.1.2.18. 4-Fluoro-*N*-(2-mercaptoethyl)benzamide (**1h**)

^1^H NMR (CDCl_3_, 400 MHz) *δ* 7.84 (dd, 2H, *J* = 8.8, 5.6 Hz, 2-H, 6-H), 7.11–7.05 (m, 3H, 3-H, 5-H, NH), 3.78 (pq, 2H, *J* = 6.4, 5.6 Hz, NCH_2_), 2.98 (t, 2H, *J* = 6.4 Hz, SCH_2_); ^13^C NMR (CDCl_3_, 100 MHz) *δ* 166.9, 164.9 (d, *J* = 251.1 Hz), 130.4 (d, *J* = 3.1 Hz), 129.5 (d, *J* = 8.8 Hz), 115.7 (d, *J* = 21.8 Hz), 39.4, 38.1; HRMS (ESI+) *m*/*z* C_9_H_11_FNOS (M + H)^+^ calcd 200.0545, obsd 200.0533.

1.1.2.19. 4-Chloro-*N*-(2-mercaptoethyl)benzamide (**1i**) (Chu, Yan, Bin, & Leilei, 2021)

^1^H NMR (DMSO-*d*_6_, 400 MHz) *δ* 8.75 (t, 1H, *J* = 5.6 Hz, NH), 7.85 (d, 2H, *J* = 8.4 Hz, Ph), 7.53 (d, 2H, *J* = 8.4 Hz, Ph), 3.55 (pq, 2H, *J* = 6.8, 5.6 Hz, NCH_2_), 2.93 (t, 2H, *J* = 6.8 Hz, SCH_2_); ^13^C NMR (DMSO-*d*_6_, 100 MHz) *δ* 166.8, 138.0, 132.7, 128.9, 128.6, 39.4, 38.2.

1.1.2.20. 4-Bromo-*N*-(2-mercaptoethyl)benzamide (**1j**) (Mano et al., 2009; Armendarez et al., 2024)

^1^H NMR (DMSO-*d*_6_, 400 MHz) *δ* 8.54 (t, 1H, *J* = 4.8 Hz, NH), 7.77 (d, 2H, *J* = 8.4 Hz, Ph), 7.65 (d, 2H, *J* = 8.4 Hz, Ph), 3.57 (pq, 2H, *J* = 6.8, 4.8 Hz, NCH_2_), 2.96 (t, 2H, *J* = 6.8 Hz, SCH_2_); ^13^C NMR (DMSO-*d*_6_, 100 MHz) *δ* 166.1, 134.2, 131.8, 129.8, 125.4, 39.5, 38.0.

1.1.2.21. 3,4-Dichloro-*N*-(2-mercaptoethyl)benzamide (**1k**)

^1^H NMR (CDCl_3_, 400 MHz) *δ* 7.95 (d, 1H, *J* = 2.4 Hz, 2-H), 7.65 (dd, 1H, *J* = 8.0, 2.4 Hz, 6-H), 7.50 (d, 1H, *J* = 8.0 Hz, 5-H), 6.94 (brt, 1H, *J* = 5.6 Hz, NH), 3.80 (pq, 2H, *J* = 6.4, 5.6 Hz, NCH_2_), 3.00 (t, 2H, *J* = 6.4 Hz, SCH_2_); ^13^C NMR (CDCl_3_, 100 MHz) *δ* 165.8, 136.3, 134.1, 133.3, 130.7, 129.4, 126.3, 39.6, 38.1; HRMS (ESI+) *m*/*z* C_9_H_10_Cl_2_NOS (M)^+^ calcd 248.9782, obsd 248.9727.

1.1.2.22. *N*-(2-Mercaptoethyl)-3,4-dimethoxybenzamide (**1l**) (Sean, Bridget, & Amedeo, 2025; Pei, James, & Ghosh, 2003)

^1^H NMR (CDCl_3_, 400 MHz) *δ* 7.45 (d, 1H, *J* = 2.0 Hz, 2-H), 7.37 (dd, 1H, *J* = 8.4, 2.0 Hz, 6-H), 6.97 (t, 1H, *J* = 5.6 Hz, NH), 6.83 (d, 1H, *J* = 8.4 Hz, 5-H), 3.90 (s, 3H, OMe), 3.87 (s, 3H, OMe), 3.79 (pq, 2H, *J* = 6.4, 5.6 Hz, NCH_2_), 2.98 (t, 2H, *J* = 6.4 Hz, SCH_2_); ^13^C NMR (DMSO-*d*_6_, 100 MHz) *δ* 167.4, 152.2, 149.3, 127.1, 119.8, 111.1, 110.7, 56.1, 56.1, 39.3, 38.4.

1.1.2.23. *N*-(2-Mercaptoethyl)-3,4,5-trimethoxybenzamide (**1m**) (Sean, Bridget, & Amedeo, 2025; Pei, James, & Ghosh, 2003)

^1^H NMR (DMSO-*d*_6_, 400 MHz) *δ* 8.64 (t, 1H, *J* = 5.6 Hz, NH), 7.18 (s, 2H, Ph), 3.81 (s, 6H, 2×Me), 3.69 (s, 3H, Me), 3.56 (pq, 2H, *J* = 6.8, 5.6 Hz, NCH_2_), 2.94 (t, 2H, *J* = 6.8 Hz, SCH_2_); ^13^C NMR (DMSO-*d*_6_, 100 MHz) *δ* 166.3, 153.1, 140.5, 129.9, 105.3, 60.6, 56.5, 39.4, 37.6.

*1.2. Simulated docking of compounds into mTYR active and allosteric sites using AutoDock Vina (Bang et al., 2018; Trott & Olson, 2010)*

The mTYR X-ray structure in the Protein Data Bank (PDB ID = 2Y9X (*Agaricus bisporus*)) was utilized for docking simulations. The PDB website (http://www.rcsb.org) was accessed on March 12, 2025. Chem3D Pro 12.0 (Cambridge, MA, USA) and ChemDraw Ultra 12.0 (Cambridge, MA, USA) were used to prepare respective 3-dimensional (3D) and 2D ligand (derivatives **1h**, **1k**, and **1m** and KA) structures. 3D candidate ligands were docked to the X-ray structure of mTYR after deletion of the original ligand (tropolone) from 2Y9X. *In silico* docking simulations were carried out using AutoDock Vina (version 1.2.0) and Chimera 1.17.3 (https://www.cgl.ucsf.edu/chimera/download.html, accessed March 26, 2025). LigandScout 4.4.8 (http://www.inteligand.com/ligandscout/download.shtml) (Inte:Ligand GmbH, Vienna, Austria) was used to identify possible chemical interactions between mTYR and each test ligand. Following validation (Supplementary Information: S79) via redocking of tropolone with 2Y9X without the original ligand, docking simulations of the test ligands with mTYR were performed.

*1.3. mTYR activity inhibition assay (Hyun, Lee, Jeong, Kim, & Choi, 2008)*

The mTYR inhibitory potency of the NMEB derivatives was evaluated in 96-well plates. mTYR (Sigma-Aldrich, St. Louis, MO, USA)-containing phosphate buffer solution (750 units/mL; 20 μL; pH 6.5) and test samples (**1a**–**1m** and KA; 10 μL; 4, 20, and 100 μM) were subsequently added to wells containing a solution (170 μL) consisting of phosphate buffer (14.7 mM [final concentration]; pH 6.5) and a substrate (l-DOPA or l-tyrosine; final concentration = 293 μM). After incubation for 30 min at 37 °C, optical density at 475 nm was measured using a VersaMax^®^ ELISA microplate (VEM) reader (Molecular Devices, San Jose, CA, USA). All experiments were performed in triplicate.

*1.4. Kinetic study experiment using mTYR (Guo et al., 2025; He, Fan, Yang, Peng, & Wang, 2023)*

Kinetic assays were conducted using 96-well plates. mTYR-containing phosphate buffer solution (pH 6.5; 150 units/mL; 20 μL) and test samples (**1h**, **1k**, and **1m**; 10 μL; final concentrations: 0, 12.5, 25, and 50 μM for **1h** and **1k**, and 0, 5, 10, and 20 μM for **1m**) were added to wells containing a solution (170 μL) consisting of phosphate buffer (final concentration = 14.7 mM; pH 6.5) and a substrate (l-DOPA; final concentrations = 1, 2, 4, 8, and 16 mM). For 30 min, the optical density of each well at 475 nm was measured every 5 min with a VEM reader. All experiments were performed in triplicate. Dixon and Lineweaver-Burk plots were prepared to analyze mTYR kinetics in the presence of NMEB derivatives.

*1.5. Copper(II) ion-chelation ability assay (Joompang et al., 2023)*

Copper(II) ion-chelation ability was assessed in 96-well plates. An aqueous CuSO_4_ solution (10 μL; 6.27 mM) and an aqueous pyrocatechol violet (SEJIN CI Co., Seoul, Korea) solution (6 μL; 4 mM) were mixed in AcOH-AcONa buffer (280 μL; pH 6.0; 50 mM) with test samples (**1a**–**1m** and KA; 10 μL [final concentration = 100 μM]) in microplate wells. After 20 min of incubation at 21 °C, each well’s optical density at 632 nm was measured with a VEM reader. Copper(II) ion-chelating activity (%) was determined utilizing the following equation: [(OD_c_ − OD_t_)/OD_c_] × 100, where OD_t_ and OD_c_ represent respective test sample and control absorbances. All experiments were performed in triplicate.

*1.6. Cell culture*

Dulbecco’s modified Eagle’s medium containing 100 U/mL streptomycin–penicillin solution and fetal bovine serum (10%) was utilized to culture B16F10 and HaCaT cells. Reagents and cells were obtained from Thermo Fisher Scientific Inc. (Waltham, MA, USA) and Korean Cell Line Bank (Seoul, Korea), respectively. Cells were cultivated at 37 °C in a humidified environment with 5% CO_2_.

*1.7. Melanin formation inhibition assay in B16F10 cells (Promden et al., 2018)*

NMEB derivatives’ ability to inhibit melanin formation was assessed in 6-well plates. B16F10 cells, seeded at 5 × 10^3^ cells/well, were cultivated for 24 h. NMEB derivatives **1a**–**1m** (20 μM) in the preliminary experiment and NMEB derivatives **1e**–**1k** (5, 10, and 20 μM) in the thorough follow-up investigation were added to cultivated cells for 1 h, followed by incubation of cells with stimulators (200 μM IBMX plus 1 μM α-MSH) for 72 h at 37 °C in an atmosphere containing 5% CO_2_. KA (20 μM) was utilized as a positive control in both experiments. Cells were washed with Dulbecco’s phosphate-buffered saline (DPBS), detached from the bottom of each well in fresh DPBS using a scraper, and centrifuged at 4 °C at 10,000 *g* for 10 min. Pellets were lysed with NaOH solution (1N; 100 μL/well) and placed at 60 °C for 30 min. Melanin was quantitated by measuring absorbance of melanin released from cells at 405 nm using a VEM reader. All data were normalized to total protein concentration in each pellet, which was determined using a Pierce^®^ bicinchoninic acid (BCA) protein assay kit (Sigma-Aldrich Co., St. Louis, MO, USA). Melanin content (%) = 100 × OD_t_/OD_c_, where OD_t_ and OD_c_ are the absorbances of the test and control samples, respectively. All experiments were performed in triplicate.

*1.8. Cell viability in B16F10 and HaCaT cells (Moon et al., 2022)*

Impact of NMEB derivatives on cell viability was assessed in 96-well plates. B16F10 and HaCaT cells were seeded at respective densities of 4,000 and 10,000 cells/well, and cultivated at 37 °C. After 24 h of incubation, derivatives **1a**–**1m** were administered to cells at 5, 10, and 20 μM. Following respective 72 and 24 h of incubation at 5% CO_2_ and 37 °C for B16F10 and HaCaT cells, a 10 μL aliquot of EZ-Cytox solution (DoGenBio, Seoul, Korea) was added to each well, and cells were again incubated for 2 h under the same conditions. Optical density of each well at 450 nm was then measured using a VEM reader to determine each derivative’s cytotoxicity. All experiments were performed in triplicate.

*1.9. cTYR activity assay in B16F10 cells (S. H. Yang et al., 2017)*

NMEB derivative impacts on cTYR activity were assessed in 6-well plates. B16F10 cells, seeded at 4,000 cells/well, were cultivated for 24 h. NMEB derivatives **1e**–**1k** (20, 10, and 5 μM) and KA (20 μM) were administered to the cultivated cells for 1 h, followed by stimulators under the same incubation conditions. Following 72 h of incubation, cells were washed with DPBS and lysed with lysis buffer (100 μL) composed of 50 mM phosphate buffer (90 μL), 1% Triton X-100 (5 μL), and 1 mM phenylmethylsulfonyl fluoride (5 μL). After being placed at −80 °C for 1 h, cells were defrosted to recover cell lysates. Centrifugation of the cell lysates at 10,000 *g* and 4 °C for 10 min afforded supernatants. In a 96-well plate, l-DOPA (2 mg/mL; 20 μL) was dispensed to each well containing the supernatants (80 μL), and each well’s absorbance was measured at 475 nm every 10 min during a 30-min incubation period. A Pierce^®^ BCA protein assay kit was used to determine total protein in each lysate to permit normalization. All experiments were performed in triplicate.

*1.10. Zebrafish larval pigmentation inhibition assay (Chen et al., 2019; Kusio-Targońska et al., 2025)*

The Zebrafish Disease Modeling Center at Chungnam National University in Daejeon, Korea, provided zebrafish (*Danio rerio*) embryos. These embryos were raised at 28 °C in an E3-MB solution until dechorionation using Pronase^®^ (Sigma-Aldrich Co., St. Louis, MO, USA) at 24 hpf. The E3-MB solution was prepared by mixing an E3 solution (containing 4 mg MgSO_4_, 1.3 mg KCl, 3.7 mg CaCl_2_, and 29.2 mg NaCl in 100 mL water) with methylene blue (final concentration: 0.001%). After a 4 h incubation in E3 solution at 28 °C, six zebrafish embryos in each well of a 48-well plate were exposed to NMEB derivatives (**1a**–**1m** [0.1 mM] for the preliminary experiment and **1a** and **1e** [0.025 and 0.1 mM] for the main experiment) or 20 mM KA. Zebrafish larvae lateral and dorsal views were photographed using an SMZ745T microscope (Nikon, Tokyo, Japan) to determine pigmentation levels at 76 hpf. The NIH Office of Laboratory Animal Welfare (OLAW) considers fish species to be “live vertebrate animals” at “hatching.” Although this is an imprecise stage in zebrafish, OLAW considers zebrafish hatching to occur at 72 hours/3 days post-fertilization. Since we completed the zebrafish experiments within 72 hours of fertilization, we did not need approval or accreditation numbers for zebrafish experiments from the Animal Ethics Committee.

*1.11. Potato juice browning assay (Huang et al., 2025; Xu, Li, Mo, Zou, & Zhao, 2022)*

Potato juice was prepared from potatoes (*Solanum tuberosum*) purchased from a supermarket in Busan, Korea, in May 2025. The potatoes (approximately 130 g) were washed with water, peeled, cut, and ground using a Tefal blender (Blend Force Glass BL317166, France). To obtain clear potato juice, the ground potato extract was centrifuged twice: first using a Labogene 416 centrifuge (4,000 × *g*; 15 mL tubes) at 19 °C for 10 min, and then using a Labogene 1730R centrifuge (10,000 × *g*; 2 mL tubes) at 19 °C for 10 min.

In each well of a 96-well microplate, an aliquot (90 µL) of potato juice was combined with 10 µL of a test sample (0.4 mM of NMEB derivatives **1a**–**1m** or KA). A VEM reader set to 405 nm was used to measure potato juice browning intensity spanning a 24 h incubation at 37 °C.

*1.12. In situ cTYR activity assay (Newton, Cook, Roberts, Leonard, & Sturm, 2007; Park et al., 2020)*

As in the cTYR activity experiment, NMEB derivatives **1e**–**1k** (20 μM) were administered to B16F10 cells for 1 h, followed by exposure to stimulators and cultivation for 72 h. Cultured cells were fixed with paraformaldehyde (4%) for 4 min and permeabilized with Triton X-100 (0.1%) for 3 min. B16F10 cells were treated with l-DOPA solution (2 mM; 500 μL) for 2 h in each well of a 24-well plate. *In situ* cTYR activity was assessed using 20 μM KA. Stained cells were photographed using a digital camera (5PM, Motic, Hong Kong) attached to a Nikon inverted microscope (Tokyo, Japan).

*1.13. In silico ADMET prediction*

To predict the physicochemical properties, medicinal chemistry friendliness, and pharmacokinetic profiles (absorption, distribution, metabolism, excretion, and toxicity) of the NMEB derivatives **1k** and **1m**, the web-based tool ADMETlab 3.0 (https://admetlab3.scbdd.com/) was utilized. The chemical structures of the derivatives were converted into SMILES strings and submitted to the platform. Key parameters including aqueous solubility (logS), lipophilicity (logP), Caco-2 permeability, plasma protein binding, cytochrome P450 (CYP) substrate/inhibitor specificity, clearance (CL_plasma_), half-life (T_1/2_), and toxicological endpoints (hERG blockage, AMES toxicity, and cytotoxicity against A549 and Hek293 cells) were calculated and analyzed.

*1.14. Statistical analyses*

GraphPad Prism 5 (La Jolla, CA, USA) was used to determine significance between groups. Results are reported as means ± SEM. To determine significance, one-way analysis of variance with the Newman-Keuls test was used (*p* < 0.05). All experiments were carried out independently and in triplicate.

**References**

Armendarez, O. J., Parizek, G. C., Augustine, R. J., et al. (2024). Preparation of poly(3-hexylthiophene) conjugated polymer brush films from amine-terminated surfaces. *Journal of Polymer Science, 62*(23), 5384–5397. doi:<https://doi.org/10.1002/pol.20240377>

Bang, E., Noh, S. G., Ha, S., et al. (2018). Evaluation of the Novel Synthetic Tyrosinase Inhibitor (Z)-3-(3-bromo-4-hydroxybenzylidene)thiochroman-4-one (MHY1498) In Vitro and In Silico. *Molecules, 23*(12). doi:10.3390/molecules23123307

Chen, Y.-M., Su, W.-C., Li, C., Shi, et al. (2019). Anti-melanogenesis of novel kojic acid derivatives in B16F10 cells and zebrafish. *International Journal of Biological Macromolecules, 123*, 723–731. doi:<https://doi.org/10.1016/j.ijbiomac.2018.11.031>

Chu, C., Yan, W., Bin, L., and Leilei, D. Reagent and method for determining total amount of sesquiterpene lactone containing α,β-unsaturated ketone in Feverfew. CN113416160 A, 2021.

El-Gendy, B. E.-D. M., Ghazvini Zadeh, E. H., Sotuyo, A. C., Pillai, G. G., and Katritzky, A. R. (2013). α-Substitution Effects on the Ease of S→N-Acyl Transfer in Aminothioesters. *Chemical Biology & Drug Design, 81*(5), 577–582. doi:<https://doi.org/10.1111/cbdd.12092>

Guo, S.-H., Zhang, Z., Zuo, X., et al. (2025). Synthesis and evaluation of the antioxidant, anti-tyrosinase and anti-browning activities of N-caffeoyl amino acid methyl esters. *LWT, 217*, 117409. doi:<https://doi.org/10.1016/j.lwt.2025.117409>

He, M., Fan, M., Yang, W., Peng, Z., and Wang, G. (2023). Novel kojic acid-1, 2, 4-triazine hybrids as anti-tyrosinase agents: Synthesis, biological evaluation, mode of action, and anti-browning studies. *Food Chemistry, 419*, 136047. https://doi.org/10.1016/j.foodchem.2023.136047

Huang, J., Li, M., Han, C., et al. (2025). Structural and mechanistic insights into the anti-tyrosinase, anti-melanogenesis, and anti-browning effect of proanthocyanidins from seed coats of Acer truncatum Bunge. *International Journal of Biological Macromolecules, 284*, 138246. doi:<https://doi.org/10.1016/j.ijbiomac.2024.138246>

Hyun, S. K., Lee, W.-H., Jeong, D. M., Kim, Y., and Choi, J. S. (2008). Inhibitory Effects of Kurarinol, Kuraridinol, and Trifolirhizin from <i>Sophora flavescens</i> on Tyrosinase and Melanin Synthesis. *Biological and Pharmaceutical Bulletin, 31*(1), 154–158. doi:10.1248/bpb.31.154

Joompang, A., Anwised, P., Klaynongsruang, S., et al. (2023). Rational design of an N-terminal cysteine-containing tetrapeptide that inhibits tyrosinase and evaluation of its mechanism of action. *Current Research in Food Science, 7*, 100598. doi:<https://doi.org/10.1016/j.crfs.2023.100598>

Jung, H. J., Park, H. S., Kim, H. J., et al. (2024). Design, Synthesis, and Anti-Melanogenic Activity of 2-Mercaptomethylbenzo[d]imidazole Derivatives Serving as Tyrosinase Inhibitors: An In Silico, In Vitro, and In Vivo Exploration. *Antioxidants, 13*(10), 1248. Retrieved from <https://www.mdpi.com/2076-3921/13/10/1248>

Kollar, L., Takacs, A., Molnar, C., Kovacs, A., Mika, L. T., and Pongracz, P. (2023). Palladium-Catalyzed Selective Amino- and Alkoxycarbonylation of Iodoarenes with Aliphatic Aminoalcohols as Heterobifunctional O,N-Nucleophiles. *Journal of Organic Chemistry*, 88, 5172–5179. DOI: 10.1021/acs.joc.2c02712

Kusio-Targońska, K., Kosheva, N., Wojtanowski, K. K., Gaweł-Bęben, K., Beis, D., and Kukula-Koch, W. (2025). Tyrosinase Inhibitors Among Flora of Lubelskie Region—Application of Bio-Chromatographic Approach and Zebrafish Model in Bioactivity Screening of Plant Material. *Molecules, 30*(9), 1979. https://doi.org/10.3390/molecules30091979

Li, H., Yang, N., Xiong, L., and Wang, B. (2021). Design, Synthesis and Biological Evaluation of Novel Thienylpyridyl- and Thioether-Containing Acetamides and Their Derivatives as Pesticidal Agents. *Molecules, 26*(18), 5649. https://doi.org/10.3390/molecules26185649

Mano, N., Aoki, S., Yamazaki, T., et al. (2009). Analysis of phosphorylated peptides by double pseudoneutral loss extraction coupled with derivatization using N-(4-Bromobenzoyl) aminoethanethiol. *Analytical chemistry, 81*(22), 9395–9401. https://doi.org/10.1021/ac9017988

Marichev, K. O., Garcia, E. C., Bhowmick, K. C., Wherritt, D. J., Arman, H., and Doyle, M. P. (2017). Highly selective acylation of polyamines and aminoglycosides by 5-acyl-5-phenyl-1,5-dihydro-4H-pyrazol-4-ones. *Chemical Science, 8*(10), 7152–7159. doi:10.1039/C7SC03184J

Matthies, D., Siewers, S., and Vogt, A. (1990). Reactivity of the C-arylglycine structure. II. Synthesis and properties of the acylation products of 3-(acylamino)-5-methyl-2(3H)-benzofuranones. *Chemiker-Zeitung*, 114(9), 283–287.

Moon, K. M., Yang, J. H., Lee, M. K., et al. (2022). Maclurin Exhibits Antioxidant and Anti-Tyrosinase Activities, Suppressing Melanogenesis. *Antioxidants (Basel, Switzerland), 11*(6). doi:10.3390/antiox11061164

Newton, R. A., Cook, A. L., Roberts, D. W., Leonard, J. H., and Sturm, R. A. (2007). Post-transcriptional regulation of melanin biosynthetic enzymes by cAMP and resveratrol in human melanocytes. *J Invest Dermatol, 127*(9), 2216–2227. doi:10.1038/sj.jid.5700840

Park, J., Jung, H., Jang, B., Song, H.-K., Han, I.-O., and Oh, E.-S. (2020). D-tyrosine adds an anti-melanogenic effect to cosmetic peptides. *Scientific Reports, 10*(1), 262. doi:10.1038/s41598-019-57159-3

Pei, Y., James, I. W., and Ghosh, S. S. Compounds for inhibiting insulin secretion and methods related thereto. WO2003066035A2, 2003. Available online: https://patents.google.com/patent/WO2003066035A2/en?oq=WO2003066035A2

Petersson, M. J., Jenkins, I. D., and Loughlin, W. A. (2009). The use of phosphonium anhydrides for the synthesis of 2-oxazolines, 2-thiazolines and 2-dihydrooxazine under mild conditions. *Organic & Biomolecular Chemistry, 7*(4), 739–746. DOI:10.1039/b818310d

Promden, W., Viriyabancha, W., Monthakantirat, O., Umehara, K., Noguchi, H., and De-Eknamkul, W. (2018). Correlation between the Potency of Flavonoids on Mushroom Tyrosinase Inhibitory Activity and Melanin Synthesis in Melanocytes. *Molecules, 23*(6), 1403. https://doi.org/10.3390/molecules23061403

Sean, M. B., Bridget, K. W., and Amedeo, V. Compounds for inhibiting insulin secretion and methods related thereto. WO 03/066035 A2, 2003, Available online: https://patents.google.com/patent/WO2018175324A1/en

Sirgamalla, R., Kommakula, A., Banoth, S., Dharavath, R., Adem, K., P, M., and Boda, S. (2018). Synthesis of Amides from Aliphatic Acids and Amines by using of I2/TBHP at Room Temperature. *ChemistrySelect, 3*(4), 1062–1065. doi:<https://doi.org/10.1002/slct.201702286>

Trott, O., and Olson, A. J. (2010). AutoDock Vina: Improving the speed and accuracy of docking with a new scoring function, efficient optimization, and multithreading. *Journal of computational chemistry, 31*(2), 455–461. doi:<https://doi.org/10.1002/jcc.21334>

Xu, H., Li, X., Mo, L., Zou, Y., and Zhao, G. (2022). Tyrosinase inhibitory mechanism and the anti-browning properties of piceid and its ester. *Food Chemistry, 390*, 133207. doi:<https://doi.org/10.1016/j.foodchem.2022.133207>

Yang, S. H., Tsatsakis, A. M., Tzanakakis, G., et al. (2017). Soyasaponin Ag inhibits α‑MSH‑induced melanogenesis in B16F10 melanoma cells via the downregulation of TRP‑2. *Int J Mol Med, 40*(3), 631–636. doi:10.3892/ijmm.2017.3061
